# Study of impacts of two types of cellular aging on the yeast bud morphogenesis

**DOI:** 10.1371/journal.pcbi.1012491

**Published:** 2024-09-30

**Authors:** Kevin Tsai, Zhen Zhou, Jiadong Yang, Zhiliang Xu, Shixin Xu, Roya Zandi, Nan Hao, Weitao Chen, Mark Alber

**Affiliations:** 1 Department of Mathematics, University of California, Riverside, California, United States of America; 2 Interdisciplinary Center for Quantitative Modeling in Biology, University of California, Riverside, California, United States of America; 3 Department of Molecular Biology, School of Biological Sciences, University of California, San Diego, California, United States of America; 4 Department of Molecular, Cell and Systems Biology, University of California, Riverside, California, United States of America; 5 Applied and Computational Mathematics and Statistics Department, University of Notre Dame, Notre Dame, Indiana, United States of America; 6 Zu Chongzhi Center for Mathematics and Computational Sciences, Duke Kunshan University, Kunshan, Jiangsu, China; 7 Department of Physics and Astronomy, University of California, Riverside, California, United States of America; 8 Biophysics Graduate Program, University of California, Riverside, California, United States of America; 9 Mathematical Institute, Leiden University, Leiden, The Netherlands; University of Tennessee Health Science Center College of Medicine Memphis, UNITED STATES OF AMERICA

## Abstract

Understanding the mechanisms of the cellular aging processes is crucial for attempting to extend organismal lifespan and for studying age-related degenerative diseases. Yeast cells divide through budding, providing a classical biological model for studying cellular aging. With their powerful genetics, relatively short cell cycle, and well-established signaling pathways also found in animals, yeast cells offer valuable insights into the aging process. Recent experiments suggested the existence of two aging modes in yeast characterized by nucleolar and mitochondrial declines, respectively. By analyzing experimental data, this study shows that cells evolving into those two aging modes behave differently when they are young. While buds grow linearly in both modes, cells that consistently generate spherical buds throughout their lifespan demonstrate greater efficacy in controlling bud size and growth rate at young ages. A three-dimensional multiscale chemical-mechanical model was developed and used to suggest and test hypothesized impacts of aging on bud morphogenesis. Experimentally calibrated model simulations showed that during the early stage of budding, tubular bud shape in one aging mode could be generated by locally inserting new materials at the bud tip, a process guided by the polarized Cdc42 signal. Furthermore, the aspect ratio of the tubular bud could be stabilized during the late stage as observed in experiments in this work. The model simulation results suggest that the localization of new cell surface material insertion, regulated by chemical signal polarization, could be weakened due to cellular aging in yeast and other cell types, leading to the change and stabilization of the bud aspect ratio.

## Author summary

Two aging modes are exhibited in budding yeast resulting in divergent shapes of the final buds. Through experimental data analysis, it is evident that control of growth rate and final bud size is more robust in cells with mitochondrial decline than those with nucleolar decline. A multiscale computational model was developed and used in combination with experiments to test the hypothesized mechanisms underlying the different types of budding in aging cells. Model simulations suggest that localized growth is sufficient to generate tubular budding. The bud aspect ratio can be stabilized by regulating chemical signals with an expanding polarization site or a decline in the rate of new cell surface material insertion. These proposed mechanisms of morphological changes in aging yeast can exist in other cell types as well.

## Introduction

Cells can reach their replication limit and terminate further division due to the cellular aging process leading to many diseases including cancer, osteoarthritis, and atherosclerosis. Understanding the fundamental mechanisms underlying cellular aging can be beneficial in treating those age-related diseases. Cellular aging impacts many processes controlling biophysical properties and biochemical signaling dynamics in individual cells. *Saccharomyces cerevisiae*, a species of yeast, replicates in each cell cycle through a process known as budding. Cellular aging occurs in yeast after multiple cell cycles. During one cell cycle, a small protrusion is first formed at a point on the cell membrane determined by certain signaling molecules. Once the cell cycle of the mother cell approaches the end of mitosis, when the cell undergoes nuclear and cellular division, the daughter cell separates from the mother cell via cytokinesis. The yeast budding process has been studied extensively, including the governing gene regulatory network and structural changes at the subcellular level to understand asymmetric growth and cell division [1–5]. Recently, budding yeast has also been used to study cellular aging by tracking their cell cycles until they stop proliferation permanently [6–15]. Cellular changes during the aging process have been identified primarily in the mother cell [5,10,16–24].

Due to the cellular changes within mother cells as they age, the bud growth rate and overall bud shape are altered. Young yeast cells always generate symmetric and rounded bud shapes with robust final bud sizes at the time of separation from the mother cell. Different bud shapes have been observed in some mutations. For example, cells with defective actin polarization or disrupted septin polymerization or stability tend to produce elongated buds with an aspect ratio, defined as the ratio between the long axis and the short axis, greater than 1.5 [25–28]. However, a recent study shows that the elongated bud shape can also be observed as a natural outcome when yeast cells get old [6,15]. In aging yeast cells, bud development in one cell cycle has been identified to differentiate into two distinct trajectories due to either chromatin instability, labeled as Mode 1, or mitochondrial decline, labeled as Mode 2. As a result, the aged yeast cells would either produce an excessively elongated bud with a very high aspect ratio and large size or a relatively small and spherical bud in Mode 1 and Mode 2, respectively [6,7,15,29]. However, the mechanisms through which these distinct bud shapes are generated in those two aging modes remain unclear.

The budding process involves biochemical signaling dynamics coupled with mechanical changes on the cell surface. Within each cell cycle, budding begins with polarization of the signaling molecule, Cdc42, and several growth-associated proteins on the cell surface. This polarization is controlled by the interaction involving multiple proteins such as Cdc42, Cdc24, Bud3, and Rga1, as well as mechanical feedback provided by the cell wall integrity pathway [30,31] and cell geometry [32]. All these regulations will lead to a single cluster of activated Cdc42 which serves as the central regulator of cell polarity in response to spatial cues. With the stabilization of the biochemical polarization, septins are recruited to form a “septin cloud” within the Cdc42-GTP cluster [33] which consequently assembles into a ring structure called the septin ring, setting the exact bud site. Actin cables are then constructed toward the bud site from the bud tip, under the assistance of Bni1, which nucleates and elongates actin filaments, and further promotes the delivery of new cell surface materials toward the bud site. During bud formation, secreted cell wall modifying enzymes alter the crosslinking in the network of beta-3-glucan–the main building blocks of the yeast cell wall–allowing for cell wall remodeling and accommodation of new materials and crosslinking [34].

The entire budding process, starting from the polarization of Cdc42 and growth-associated protein in the G1 phase, to the abscission of the daughter cell in the M phase, usually takes approximately 90 minutes for younger cells. As the cell ages, the budding process is extended, and can take more than 300 minutes for an old cell near the end of its lifespan [6]. However, the causes of deceleration of the budding process and formation of the two distinct bud shapes remain unclear. Current theories revolve around it being related to the production and delivery rate of the cell surface materials, or the cell wall extensibility that determines the cell wall deformation. Furthermore, changes in the mechanical properties may also play a role. It has been suggested that aged yeast cells have their cell wall elasticity altered where bud scars are formed and that the cell surface usually becomes stiffer when compared to the cell surface of yeast cells with fewer bud scars [35]. Therefore, based on the recent experimental observations in [6,15] and the major components identified to be involved in the yeast budding process, we hypothesize that the delivery rate and location of new cell surface materials, under the regulation of polarized chemical signals, determine the bud growth rate and the final bud shape. Cellular aging may affect the size and maintenance of the polarization site of chemical signals, giving rise to the different modes of budding in old cells.

Many computational models have been developed to study the underlying mechanisms of the budding and similar processes which result in the formation of a protruding structure. Classical discrete tether-and-bead type models have been utilized to study the evolution of shapes ranging from the internalization of nanoparticles to the tip-extending process such as pollen tube growth, with emphasis on the effect of surface mechanical properties [36–41]. Thin shell theory has also been used to study how the cell surface mechanical properties feedback correlates with the tip-growth “shmooing”, where a nodule is extended from the cell, during the yeast mating process [30]. The interplay between the cell surface mechanical properties, surface topologies, cell surface synthesis, and subcellular processes has gained lots of attention and been studied extensively. These studies require the coupling between submodels that represent different key components of the yeast budding process. A model that incorporates cell wall elasto-plasticity, osmolarity, and turgor pressure has been developed to study the role of HOG signaling pathway in yeast osmoregulation [42]. Cdc42 polarization model has been coupled with subcellular processes to investigate how the cell geometry affects the polarization dynamics in [32] and a coarse-grained continuum model has been developed to investigate the coupled dynamics of cell polarization and morphogenesis during yeast mating [30].

Previously, we developed a 3D coarse-grained particle-based model to study the effect of changes in mechanical properties along the cell surface on the bud formation process [43]. The sphere, representing the combined cell wall and membrane of a mother yeast cell in the model, is discretized into a triangulated mesh in 3D. To model the elasticity of the modeled cell, vertices in each triangle are connected by linear springs to capture the in-plane elasticity, whereas neighboring triangles sharing a common edge are connected through bending springs to represent the out-of-plane elasticity of the cell surface, similar to the approach utilized in [44]. Moreover, the model assumes that turgor pressure remains constant throughout one cell cycle before the bud separates, based on the fact that the mother cell exhibits marginal change in size during the early stages of the budding process and the turgor pressure remains sufficiently constant in the range of 0.1–1.0 MPa under the osmotic regulation in yeast cells [45–47]. Using this computational framework, it has been shown that a higher stretching to bending stiffness ratio in the bud region than that in the mother cell is sufficient for bud emergence [48]. In this work, we extend the model by coupling the mechanical submodel with a chemical signaling submodel to study the interplay between cellular morphology and growth-associated chemical signaling distribution. The relevance of our approach lies in the ability to describe the asymmetric growth via Monte Carlo re-meshing method to accommodate drastic topological changes while demonstrating the stability of reaction-diffusion equation, describing the dynamics of growth-related chemicals, over an evolving surface mesh. Our multiscale coupled model enables a more realistic representation of the budding process at the morphological and the subcellular level, and allows us to study the budding process from bud emergence to the point before cytokinesis, especially mechanisms of the bud morphogenesis involving interplay between mechanical properties and chemical signals.

In this study, a combined experimental analysis and modeling simulation approach is used to test hypothesized mechanisms regulating cell cycle length and production of different bud shapes due to cellular aging in yeast. By measuring bud growth rate, size, and shape in experimental images obtained at different ages of yeast cells fixed in a microfluidic channel, it is found in this work that budding always follows a linear growth regardless of cell age, and the cell cycle length becomes much longer in old cells. Cells in different aging modes exhibit different robustness in maintaining the bud size even when cells are young. For old cells that give rise to tubular budding, the bud aspect ratio only increases at the early stage of each cell cycle and remains constant while the bud keeps growing until cytokinesis. By applying the calibrated model developed previously [43] and coupling it with the chemical signaling model of the Cdc42 pathway, we have identified that a constant rate of delivery of new cell surface materials to the bud site can achieve linear bud growth. Moreover, a tubular bud shape can only be obtained by a spatially localized insertion of new cell surface materials and not the nonhomogeneous mechanical properties over the cell surface. Simulation results also predict that the stabilized aspect ratio of tubular budding can result from the diminishing polarization of Cdc42, suggesting cellular aging may affect the maintenance of the polarizing signals.

## Results

### Experiments suggest two modes of cellular aging with different growth rates and final sizes and shapes

Bud growth of wild-type yeast has been shown to produce a linear increase in the bud surface area when the mother cell is relatively young [49]. However, the budding process of old mother cells has not been well studied. The age of a yeast cell can be determined via the number of replications of the cell. Hence, it is called the replicative age [9,50]. The assay is usually analyzed by tracking the number of daughter cells a yeast cell has produced. Alternatively, since each yeast cell has a different number of total replications, the approximated age is usually determined by the lifetime percentage (current number of replications divided by the total number of replications). It has been found that the cell cycle time is quite robust among yeast cells at a similar lifetime percentage [6].

**Fig 1:**
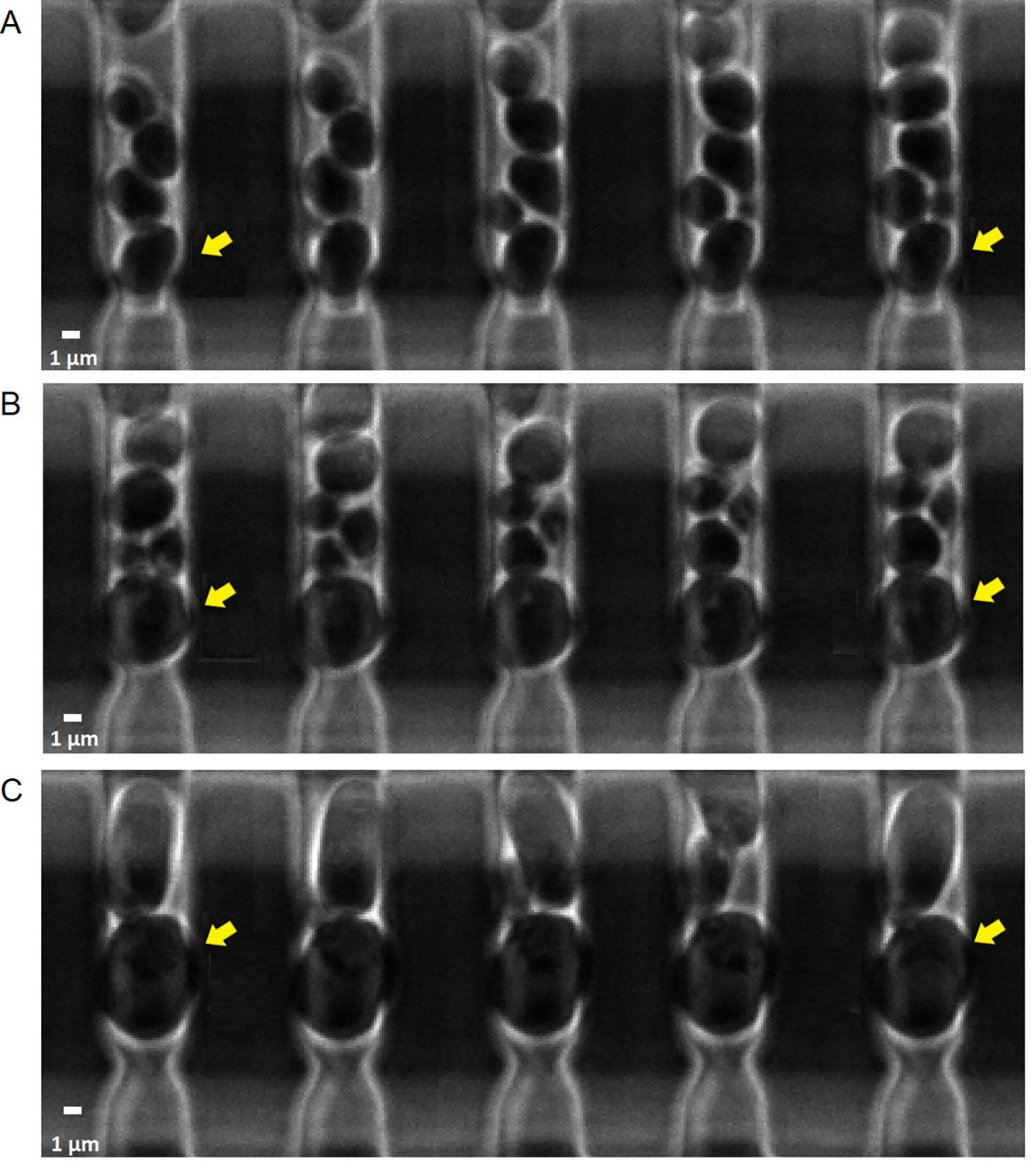
Sample experimental images depicting the budding process. The progression is oriented from left to right where the leftmost image represents the beginning of the budding process. Yellow arrows indicate the mother cells tracked. White bar on the lower left corner is the scale bar representing approximately 1*μm*. (A) A young yeast cell producing spherical buds. (B) An aged yeast cell in Mode 2 gave rise to a spherical bud. (C) An aged yeast cell in Mode 1 gave rise to a tubular bud. Time between successive snapshots is (A) 15 minutes, (B) 30 minutes, (C) 30 minutes except the rightmost image was captured after 180 minutes since the previous snapshot.

**Fig 2:**
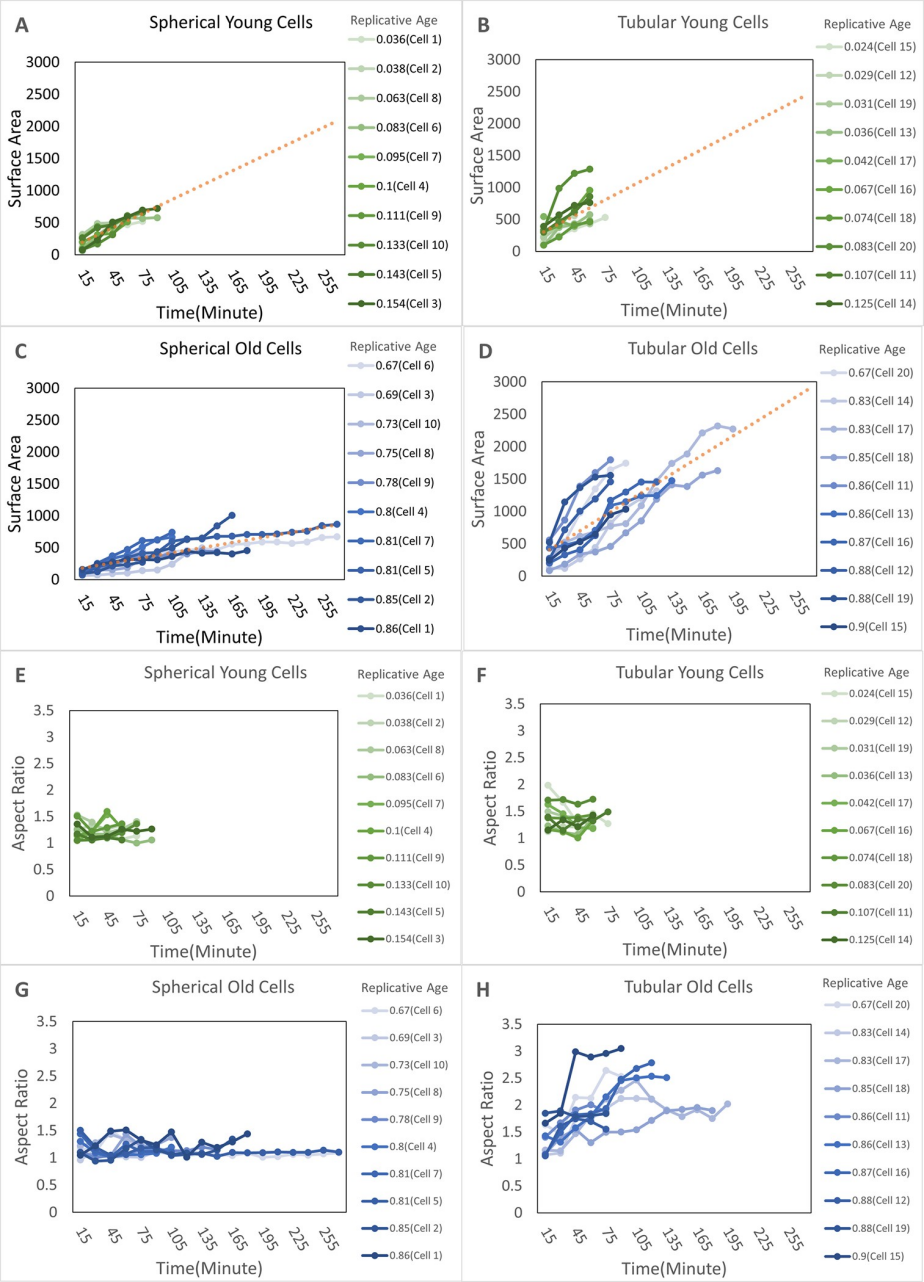
Image analysis of bud growth rate and aspect ratio of cells at different ages in experiments. (A) Time history of surface area for spherical buds from young yeast cells which still give rise to spherical buds when they are old (Mode 2). (B) Time history of surface area for spherical buds from young yeast cells which give rise to tubular buds when they are old (Mode 1). (C) Time history of surface area for spherical buds by the same mother cells as (A) when they are old. (D) Time history of surface area for tubular buds of the same mother cells as (B) when they are old. Red dot lines in (A-D) represent the linear fitted lines of experimental data shown in each figure. (E) Time history of aspect ratio for spherical buds same as (A). (F) Time history of aspect ratio for spherical buds same as (B). (G) Time history of aspect ratio for spherical buds by the same mother cells as (A) when they are old. (H) Time history of aspect ratio for tubular buds of the same mother cells as (B) when they are old.

Budding of individual yeast cells was tracked using experimental images throughout the lifetime of each cell [14] (see Materials and Methods for more details) (Fig 1). It has been shown that aging yeast cells can develop into two different bud shapes, with one more tubular, driven by nucleolar decline in Mode 1, and the other more spherical, driven by mitochondrial deterioration in Mode 2 [6]. The bud surface area was measured and the bud aspect ratio calculated for both young and old mother cells to investigate the bud shape formation and budding growth rate as cells age in different modes (Fig 2).

### Spherical budding reaches similar final bud size at different ages with growth rate reduced in older cells

We first focus on cells that always give rise to spherical budding regardless of cell age, i.e. Mode 2. The budding process is initiated fast on young mother cells and the bud surface area undergoes a linear growth (Fig 2A), which is consistent with the observation in previous literature [49]. Among all different samples, the growth rate is similar, and similar final sizes are reached within about 75–100 minutes after bud initiation. This suggests young mother cells in Mode 2 experience very similar cell cycles and generate similar bud shapes. As mother cells age, the cell cycle becomes significantly longer. Some buds grow much more slowly after bud initiation and some old mother cells take much longer to initiate the budding process (Fig 2C). However, the final bud size obtained remains similar to those obtained by young mother cells. The aspect ratio of buds stays close to 1 no matter how old mother cells are, and it also remains constant during budding for both young and old mother cells (Fig 2E and 2G). In summary, this data indicates that in Mode 2, older mother cells have much longer cell cycles than young cells, while maintaining the spherical bud shape throughout the budding process and reaching a robust final bud size.

### Tubular budding of aging cells shows large variation in both bud size and growth rate

In contrast, cells in Mode 1 have very different behavior even when they are young. Although young mother cells in this aging mode also generate spherical buds, the growth rate varies a lot from sample to sample (Fig 2B). Some buds grow linearly at rates similar to cells in Mode 2. Others grow much faster with short cell cycles or grow much more slowly. Although most samples still grow linearly, the variation among young cells in Mode 1 is much larger than in Mode 2. Similar behavior is also observed on the bud aspect ratio (Fig 2F). The variation of aspect ratio among samples is large, remaining constant within individual cell cycles and the overall average is still close to 1. This indicates a spherical shape is maintained in individual cell cycles for young mother cells in Mode 1.

As mother cells in Mode 1 get older, on average, bud initiation becomes faster (Fig 2D) and bud shape becomes asymmetric right after the bud initiation (Fig 2H). For most of the samples we collect, buds still grow linearly, and the bud aspect ratio increases rapidly only during the early stage followed by a saturation, while the bud keeps growing. Large variation is observed in the aspect ratio from sample to sample (Fig 2H). All tubular buds collected reach much larger final sizes than those generated by young cells and the cell cycle of aging cells becomes much longer. In comparison to cells in Mode 2, which always generate spherical buds of similar size at different ages, the final size of tubular budding shows a large variation among samples (Fig 2A–2D). This indicates a less effective size control mechanism governing aged cells in this mode. The growth rate of tubular budding also varies significantly from sample to sample, but the average rate is similar to that in young cells (Fig 2B and 2D).

Based on the comparison between young and old mother cells in those two modes, we notice that the yeast budding behavior starts to diverge in terms of the shape’s robustness in young cells. However, the average bud size and shape remain similar for the two aging modes. As mother cells age, the divergent behavior becomes more visible. Buds with different sizes and shapes are generated, while the bud growth remains linear in both modes (Fig 2A–2D). Such linear growth of the bud surface area, observed in both spherical and tubular budding, justifies a model assumption that new cell surface materials are delivered and inserted into the bud region periodically at a roughly constant rate throughout the budding process resulting in linear growth. Moreover, as shown in experiments, for both spherical and tubular budding modes, this cell surface material insertion rate changes as cells age.

We develop a computational framework based on the model developed in [43], coupled with polarizing chemical signal Cdc42, to investigate the mechanism underlying the growth rate change and bud shape formation in different modes due to cellular aging. We assume the cell surface material insertion rate is constant and the growth event is introduced periodically in the model as described in detail in Materials and Methods. Furthermore, we calibrate the increase in the bud surface area per growth event in the model using the temporal bud growth rate obtained from experiments. Such calibration on the bud growth trajectory is implemented for both young and older mother cells, as well as for spherical budding and tubular budding (SeeS1 Text). We also assume the new cell surface materials are only added to the polarization region of Cdc42 within the bud surface. We apply this coupled model to test whether nonhomogeneous mechanical properties or nonhomogeneous cell surface growth can give rise to the tubular bud shape as observed in Mode 1 in experiments.

### Model simulations predict the mechanism underlying different bud shape formation due to cellular aging

Recently, it has been noticed that wild-type yeast cells could undergo distinct aging-driven differentiation processes [6,15]. One cell life cycle will progress toward either nucleolar decline with a more tubular bud shape, or mitochondrial decline with a more spherical bud shape [6,7]. Our new experimental data suggests those two aging trajectories diverge at a young age. The mechanism underlying different bud shape formation due to cellular aging remains unclear. During the budding process, some chemical signaling is polarized on the cell membrane, rearranging the actin cables to follow a similar polarized distribution inside the cell [34,51–54]. Consequently, new materials are delivered along the polarized actin cables to the cell surface, leading to bud growth. Such new material insertion in the bud surface may also change the mechanical properties locally, or inversely, the local mechanical properties may affect the new material insertion. Extensive studies have been conducted to understand the dynamics of those critical signaling networks and the mechanism to establish cell polarization [2,30,34,55,56]. By coupling the mechanical model we developed previously in [43] with the chemical signaling model describing one of the major signaling pathways in yeast, Cdc42, we study the mechanism underlying different bud shape formation due to cellular aging.

### Growth restricted to the bud tip leads to tubular budding

**Fig 3:**
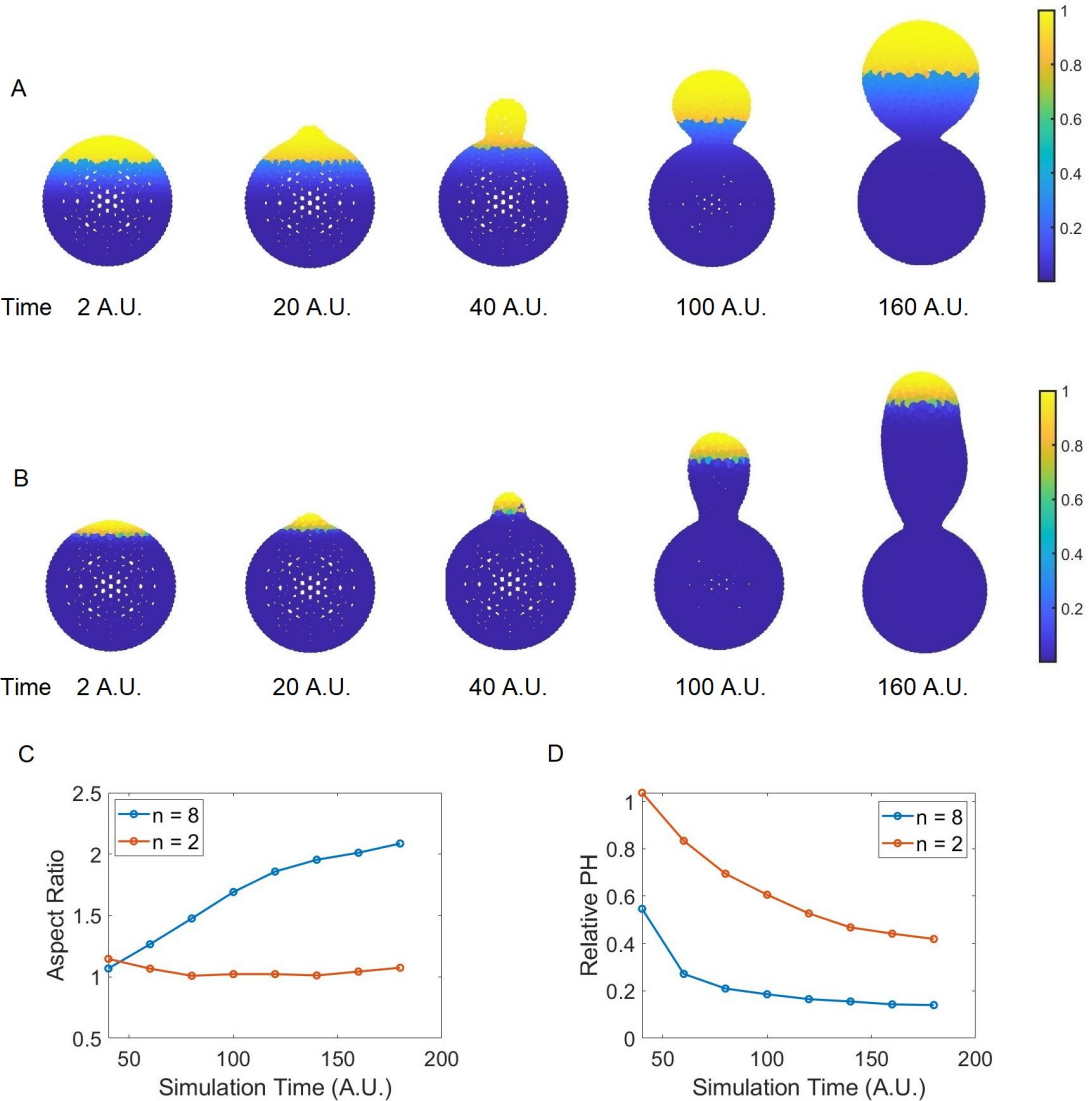
Sample simulations where the mechanical model is coupled with a chemical signaling model which determines the location of cell surface material insertion. Polarization shown here is a time-dependent process. Growth, restricted to the bud site, characterized by material insertion is introduced based on the local concentration. Such concentration must exceed a specific threshold value. Yellow color represents locations where material insertion is possible, while blue color represents locations where the chemical concentration is below a threshold value, 0.8·*Conc*_*max*_. Here *Conc*_*max*_ is the maximal value of concentration over the whole cell surface. (A) depicts a case where the polarization is achieved by setting *n* = 2. Here *n* is the constant determining the sharpness of the polarization. (B) depicts a case when *n* = 8. The more polarized the signal directing material insertion is, the more elongated the resulting bud becomes. (C) Corresponding aspect ratio during bud formations. (D) Corresponding polarization height (PH), based on the concentration threshold described above, during bud formations.

In our chemical submodel, we utilize a spatial signaling gradient *u* to represent the gradient of some molecular stimuli and to determine a preferred polarization site of intracellular signaling molecules including Cdc42. This allows us to control the local concentration of Cdc42 and its associated growth proteins in a simplified way (see Materials and Methods). The gradient *u* is described by the following formula: ${u=u}_{min}+(u_{max}-u_{min})\cdot ((H_{T}-H_{min})/H_{total}{)}^{n}$, where *H*_*T*_−*H*_*min*_ represents the distance between the z-coordinate of the center of a triangle in the bud mesh to the z-coordinate of the bottom of the mother cell. *H*_*total*_ is the distance from the bud tip to the bottom of the mother cell, and *n* a scaling factor controlling the sharpness of the gradient (Fig C(C) in S1 Text). The growth region within the bud surface is chosen to be the area with a Cdc42 concentration greater than some threshold value. This threshold value is selected such that the size of the bud growth region and the dynamics of the bud growth are similar to those observed in experiments. To quantify the region of cell wall material insertion, i.e. the growth region, we utilize the metric “polarization height”. Polarization height (PH) is the distance from the z-coordinate of the tip of the bud (daughter cell) to the z-coordinate of the edge of the region where chemical concentration is at least 0.8*Conc*_*max*_. Here *Conc*_*max*_ is the maximum chemical concentration on the bud surface. Relative PH is calculated as *PH*/*PH*_*total*_, where *PH*_*total*_ is the distance from the z-coordinate of the bud tip to the average z-coordinate of all nodes on the septin ring, representing the bud neck. The result describes an estimation of the portion of the bud in height where the chemical concentration is above the aforementioned threshold value, representing the polarization region. Varying the sharpness of the gradient *u* in the simulation will lead to a change in the growth region. We ran the simulations with *n* = 2 and *n* = 8 respectively. With *n* = 2, due to the almost linear spatial gradient *u*, the distribution of the signaling molecule was less polarized and its concentration over the entire bud was higher than the threshold value at the early stage (Fig 3A). Therefore, new materials were inserted almost evenly over the entire bud surface, and a spherical budding was generated (Fig 3A). With *n* = 8, the spatial gradient *u* was decaying rapidly from its maximum at the bud tip to the rest of the bud surface, and therefore the signaling molecule was more concentrated near the tip. By using the same threshold value, the growth region in this case, became much smaller, and new materials were only inserted into a subregion around the tip, giving rise to a tubular bud (Fig 3B). Therefore, our coupled model can generate both spherical budding and tubular budding by employing different gradients *u* as the spatial cue. For a shallower spatial cue, the growth region is large relative to the bud size at the early stage (Fig 3D) and a spherical bud is obtained. For a steeper spatial cue, since the growth region determined by the chemical signal is sufficiently small relative to the bud size throughout the budding process, a tubular bud is obtained with an increasing aspect ratio (Fig 3C). Overall, this suggests restricted growth at the bud tip is sufficient to give rise to tubular budding. Cellular aging may affect the polarization of the signaling molecule through different biological processes including restricting the diffusion and activation in space or localizing the delivery and insertion of the new materials within the bud surface, such that the growth becomes more restricted to the bud tip, and generates tubular budding.

### Kinetics of stabilizing the tubular bud shape

**Fig 4:**
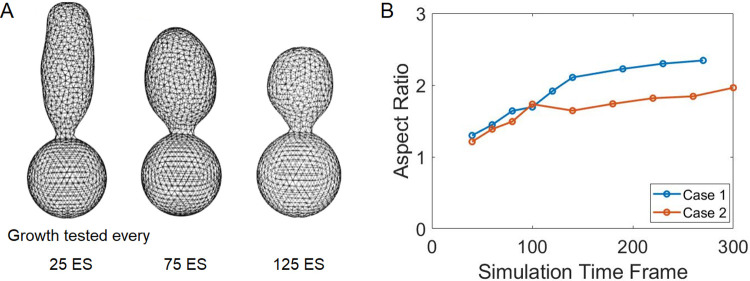
Impact of Edge Swapping (ES) on bud shape. (A) Simulations of budding with spatially biased growth and with 25, 75, or 125 ES steps between successive growth events. As the number of ES steps increases, the relaxation time extends, leading to a progressively more spherical bud shape. Note that the material insertion is confined to a small region at the bud tip. (B) Increased edge swapping steps between successive growth events can stabilize the aspect ratio of the bud shape. Case 1 and 2 represented the simulations where the edge swapping steps between successive growth events changed from 25ES to 50ES after 150 and 100 simulation time frames, respectively.

In simulations, the cell wall over the entire bud surface can rearrange itself locally and is modeled by a stochastic re-meshing approach, called edge swapping (ES) [43,57]. The goal of edge swapping is to relax the energy of the system after the addition of new nodes. To see the effect of edge swapping on the bud shape, we first test the scenario of spatially biased growth with the growth region fixed at the tip of the bud and then let the entire bud surface relax. To test the effect of the relaxation time, i.e., the number of edge swapping steps, we run the simulations with 25, 75, or 125 edge swapping steps following each growth event. The simulation results show that the bud shape is tubular when the number of edge swapping steps is small, and the bud becomes spherical when the number of edge swapping steps is large (Fig 4A). This suggests that the tubular bud shape can only be obtained when the growth time significantly surpasses the relaxation time. In other words, more materials must be added before the system reaches equilibrium to give rise to a tubular bud shape. Thus, the tubular bud shape can only be obtained under the nonequilibrium conditions, not as a structure with minimal energy, under the spatially biased growth.

As observed in experiments, the tubular budding has its aspect ratio increasing only at the early stage of one cell cycle and then it is maintained while the bud keeps growing until the end of the cell cycle (Fig 2H). This suggests that the cell wall rearrangement may become more active at the later stage of budding, which can stabilize the aspect ratio. Hence, we test the case with an increasing number of edge swapping steps between successive growth events during budding. By implementing edge swapping more frequently at the later stage of budding, the aspect ratio can be almost stabilized as the system is approaching a steady state (Fig 4B). Therefore, the stabilized aspect ratio of tubular budding in the late stage may result from a balance between the insertion of new cell surface materials and the cell wall rearrangement to relax the energy of the system.

Another hypothesis is that the spatially restricted growth might be dynamically changing during budding such that the growth is more localized at the beginning and then spreads into a larger region, under the guidance of polarized biochemical signaling molecules. In the following section, we study a time-varying growth region to understand this alternative mechanism for maintaining the aspect ratio in tubular budding.

### Reduction in polarization stabilizes the aspect ratio of tubular buds

**Fig 5:**
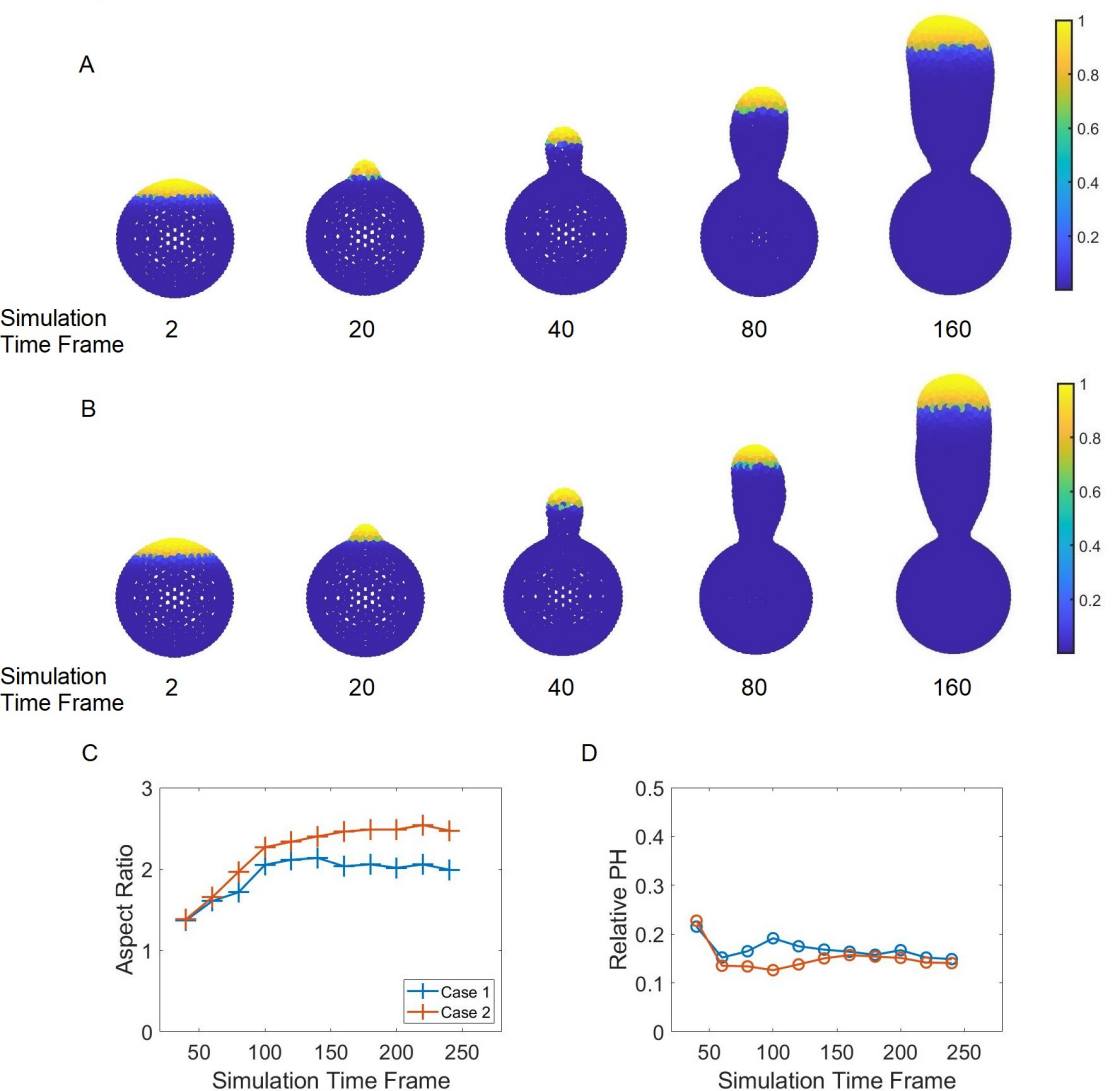
(A, B) Sample simulation snapshots demonstrating the change of the polarization pattern during bud development corresponding to Case 1 and 2 in (C, D), respectively. From left to right, each image corresponds to simulation time frame 2, 20, 40, 80 and 160, respectively. (C) Aspect ratio of the bud produced. Case 1 represents the scenario where the scaling factor *n* starts with 5 and increase to 12 in 5×10^3^ simulation time steps, and then decrease to 7 in 5×10^3^ simulation time steps. Case 2 is of the identical setup to Case 1 except that the scaling power *n* completes the reduction in 1×10^4^ simulation time steps. (D) Corresponding relative PH (relative height of the region eligible for material insertion to the height of the bud). The maintenance of the aspect ratio is directly linked to the relative PH in terms of stabilization of such values.

Experimental observations highlight a switch in the distribution of the polarizing signaling molecules, especially Cdc42, and its associated growth proteins. More specifically, Cdc42 switches from being polarized during bud emergence to a more spreading distribution within the bud, followed by localization near the bud neck at the end of one cell cycle [58]. We hypothesize that the spatially restricted growth region changes over time in a similar manner, and is determined by the biased spatial gradient *u*, which is also changing over time in the chemical signaling submodel. In particular, the parameter *n* in the formula of *u* is assumed to be a function of time, *n*(*t*). This temporally changing spatial cue is assumed to shrink in space at the early stage of one cell cycle and then expand, followed by the growth region (Fig 5A and 5B). In simulations, the aspect ratio of the bud first increases and then stays constant while the bud keeps growing (Fig 5C). The stabilization of the aspect ratio starts exactly at the time when the signal becomes less polarized. It was also shown that the size of the growth region, relative to the bud size, decreases first and then stays the same (Fig 5D). Reducing *n*(*t*) over a longer time period in the function of *u*, representing the gradient changing from being steep to shallow, maintained the aspect ratio at a higher level (case 2 in Fig 5), compared to the case with more rapidly decreasing *n*(*t*) (case 1 in Fig 5). This suggests that cellular aging in yeast may affect how long the signaling polarization can be maintained after it is established and how fast the polarized distribution vanishes.

## Discussion

In this study, a combination of experimental data analysis and a biologically calibrated multiscale computational mechano-chemical model was used to test hypothesized mechanisms underlying different modes of bud shape formation and changes in bud growth rates of aging yeast cells. Experimental data analysis has shown that cells aging in different modes have different levels of robustness in controlling bud size and growth rate even at a young age. This data was also used to calibrate the mechanical submodel developed earlier in [43], to study the necessary mechanical properties of the membrane for bud emergence. The coupled model included a novel chemical signaling submodel representing Cdc42 signal in the form of a system of reaction-diffusion equations solved on the triangular mesh on the growing 3D cellular surface. Cdc42 signal can achieve polarized distribution at the early stage of one cell cycle and is responsible for regulating the rate and location of new cell surface materials. The simulated polarized chemical signal was used in the mechanical submodel to guide the spatially biased growth. The model was calibrated to reproduce the linear growth of budding by applying a constant insertion rate of new cell surface materials within the Cdc42 polarization site. The model was first calibrated to achieve experimentally observed linear cellular growth rate. Then, it was used to test hypothesized mechanisms underlying the tubular bud shape formation and maintenance of its aspect ratio while the bud size keeps increasing at the later stage of one cell cycle.

Analysis of experimental images suggests that the budding dynamics are quite different in the two aging modes even when cells are young and despite the bud surface area increasing linearly in both modes. For spherical budding, the average bud size remains similar at different ages. However, in aging cells, the variation in average bud size among different samples becomes larger and the growth rate is reduced (Fig 2C). For tubular budding, even when cells are young and still produce spherical buds, the variation of bud size among different samples is much larger, indicating less robust bud sizes obtained in this mode (Fig 2B). Variation of bud sizes and growth rates among different samples increases significantly in aging cells with tubular budding (Fig 2D). We calibrated the mechano-chemical model to achieve a specific experimentally observed linear growth rate of budding. Model simulation results indicate that non-uniform mechanical properties of the cell surface are not sufficient to generate asymmetric bud shapes. They further predict that the spatially biased growth that may occur in aging cells is required to give rise to tubular budding. Such tubular bud shape can only be maintained under insufficient cell wall modification, computationally represented by employing a limited number of Monte Carlo cell surface remeshing on the bud surface as a nonequilibrium (Fig 4). By coupling with the dynamic polarizing chemical signal Cdc42, the model can generate more biologically relevant bud shapes with aspect ratio maintained at the later stage of tubular budding. Such maintenance relies on an expanding polarization site of Cdc42 in the late stage of one cell cycle, suggesting cellular aging may affect the maintenance of chemical signal polarization.

One possible extension of the studies described in this paper would be to consider the bud initiation guided by the polarizing biochemical signals in individual cell cycles. In our current model, the bud location was given manually as one initial condition and fixed throughout the simulation. However, it was suggested that structural components required for initial budding were delivered by actin cables whose distribution was dependent on the polarization of the signaling molecules [59,60]. However, it is still under debate how early and how quickly the biochemical signals became polarized and how this process changes during aging. The existence of the septin ring would also affect the chemical signaling distribution since it would reduce the diffusion as a physical barrier [61–63]. Furthermore, it is worth investigating the polarizing signals throughout one cell cycle in old cells experimentally. Following the polarizing biochemical signaling idea, another potential extension of the current model is to incorporate the effect of septin distribution and dynamics. At a basic level, a reservoir-type model accounting for the septin molecules within the mother cell may be introduced. This can be utilized to describe the transportation of septin molecules to the bud site and the essential formation of the septin ring, from a cluster of septin molecules, through the interplay between Cdc42 and other growth-associated molecules at the early stage of budding process. On the other hand, its potential role in the redistribution of Cdc42 at the later stages of the budding process would also be of interest.

Another interesting question to study is how the linear growth in bud surface area was achieved mechanistically. In our current model, the new material insertion rate into the cell surface is assumed to be constant, which might be an outcome of the mechanical properties and the regulations governing biochemical processes. This growth rate is maintained within one cell cycle and reduced as mother cells get older. It would be interesting to link the new material insertion rate to polarizing signals in the model to understand the mechanism.

It would also be interesting to simulate the late stage of the budding process, including the cell division, and to explore the size control mechanism as mother cells get old. We only model one cell cycle (one budding event) and the size of a mother cell does not change significantly as observed experimentally (Fig 1). Our experimental data showed that the final bud size remained similar for cells at different ages in the mode of spherical budding, whereas the final bud size varied a lot among different samples in the mode of tubular budding even when mother cells were still young. This suggests that the bud size control may not be affected by aging in spherical budding, but it becomes less robust even in young cells with tubular budding. It is known that aging cells may undergo ribosomal DNA instability, leading to dysregulation in ribosomal biogenesis involved in cell size control [10,16]. It has been observed that the polarized signaling molecules lost the polarization distribution near the tip and became concentrated near the septin ring before the cell division occurred. It was then followed by the shrinking of the septin ring, leading to the complete separation of the bud from the mother cell. Both biochemical signals and mechanical components were rearranged when the final bud size was achieved. Our mechano-chemical model could be applied to understand the underlying mechanism. By simulating the entire cell cycle of yeast budding, it would also be possible to extend the model to study the budding process with multiple generations and understand how cellular aging affected the critical biological processes dynamically to give rise to different bud shapes in daughter cells and how their lifespans are affected.

Overall, the experimental data analyzed here suggests that cells can age in two different modes with different levels of robustness in controlling bud size and growth rate from the start. This indicates that aging mechanisms may take effect in cells already at young ages and have an impact on size control. Model simulations predict potential mechanisms for bud morphogenesis during cellular aging which can be verified in future experiments on the localization and maintenance of Cdc42 polarization in aging cells. However, whether and how such morphological changes are related to lifespan control remains unclear. Addressing this question requires technological advancement that enables lifespan tracking of daughter cells as well as mother cells from the same experiments. In future studies, when new technologies and data become available, it will be interesting to investigate the functional role of age-dependent morphological changes in lifespan control. The mechano-chemical model developed in this study can also be adapted to study the aging of other cell biology systems in the context of cell growth and cell division.

## Materials and methods

Experimental procedures and techniques can be found in S1 Text. A detailed description of the mechanical model and mechano-chemical coupled model is also provided in S1 Text, including the modeling calibration, numerical method, and additional modeling analysis.

### Mechanical submodel

The mechanical submodel utilized in this study is similar to the one developed in [36]. The mother cell is set up as a sphere with triangulated mesh which is a simplified representation of the elastic network of the yeast cell surface. A region on the cell surface is designated as the budding region enclosed by the septin ring. Within the mesh, the motion of each node is described by $c{\dot{x}}_{i}(t)=-\nabla_{x_{i}}(E_{total})+F_{turgor,x_{i}}$, where *c* is the friction coefficient depicting the viscosity of the cell surface, $\nabla_{x_{i}}$ represents the gradient with respect to *i*^*th*^ node coordinate, *E*_*total*_ represents the total potential energy used to model the mechanical properties of the cell surface, and $F_{turgor,x_{i}}$ is the force derived from the turgor pressure. Between any two nodes, the linear elastic interaction is described by the linear spring potential, $E_{linear}=\sum (\frac{k_{s}}{2})(L-L_{0}{)}^{2}$, where *k*_*s*_ is the spring coefficient, *L* is the current length of the spring and *L*_0_ is the equilibrium length of the spring. For the aforementioned septin ring, the segments within the ring are also described via a similar linear spring potential except the spring coefficient is scaled by 2*L*_0_^2^ instead of 2. Between any two adjacent triangles, the bending interaction is described by the cosine bending potential, $E_{bend}=\sum k_{b}(1-cos(\theta -\theta_{0}))$, where *k*_*b*_ is the bending coefficient, *θ* is the current dihedral angle between unit normal vectors of the two triangles, and *θ*_0_ is the equilibrium dihedral angle. For each triangle, the local area expansion resistance of each triangular facet is presented using the harmonic potential, $E_{area}=\sum (\frac{k_{a}}{2A_{0}})(A-A_{0}{)}^{2}$, where *k*_*a*_ is the resistance coefficient, *A* is the current area of the triangle, and *A*_0_ is the equilibrium area of the triangle. Standard Morse potential, $E_{volex}=\sum D(1-exp(-a(L-L_{0})){)}^{2}$, *L*≤*L*_0_ is also prescribed to each node for the self-avoiding property. Here *D* is the well-depth of the Morse potential with width *a*, *L* is the distance between any two non-connecting nodes, and *L*_0_ is the minimum distance between any two non-connecting nodes. The budding process is presented as a combination of cell surface expansion and cell surface rearrangement. The cell surface expansion is modeled by splitting two adjacent triangles into four triangles, namely, for a pair of adjacent triangles, a new edge is formed between the two unconnected nodes turning a two-triangles-four-nodes configuration into a four-triangles-five-nodes configuration [36]. On the other hand, the Monte-Carlo re-meshing algorithm is used to probabilistically determine if the central edge of a pair of triangles needs to “flip” to establish a new connectivity between the two previously unconnected nodes.

### Mechano-chemical computational model

In this section, we describe the coupling of different submodels in space leading to a multi-scale model of cell budding. The novelty of the chemical signaling submodel is in its implementation on a growing 2-dimensional cell surface in 3-dimensional space. Namely, a quasi-steady state solution of a system of reaction-diffusion partial differential equations representing cell signaling is numerically calculated on the deforming triangular mesh generated by the mechanical model determining cell surface. Mechanical properties and the insertion region of new cell surface materials are updated based on the spatial gradient of the chemical signal to follow a similar polarized pattern within the bud surface. This is a two-way coupling in the sense that the chemical signal impacts cellular mechanical properties and spatially biased growth, whereas the resulting bud shape provides a deforming domain for the signaling submodel. This multi-scale model is applied to study the interplay between the cellular physical properties and the biochemical cues affecting bud shape formation and maintenance.

The signaling submodel involves Cdc42 similar to (38). It is known that at the beginning of a budding cycle, Cdc42 is initially distributed almost uniformly along the cell surface and cytosol. Upon activation, multiple clusters of Cdc42 form on the cell surface and, eventually, they evolve into a single polarization site due to the self-promoting activation and competition between clusters on the acquisition of polarization-promoting microstructures (2, 27, 36). In the chemical signaling model we introduced, the competition between Cdc42 clusters is neglected. This is because the competition only happened over a short period of time and it was assumed that a spatial cue *u* determined the location of the single polarization site. In addition, we assume that the inactive Cdc42 and other growth-associated proteins were well-mixed in the cytosol at the beginning. To establish polarization on the cell surface, represented by *Γ*, we utilized the following reaction-diffusion partial differential equations studied in [64]:

$$\frac{\partial a}{\partial t}=D\Delta_{\Gamma }a+\frac{k_{0}}{1+(\beta u{)}^{-q}}+\frac{k_{1}}{1+(\gamma pa{)}^{-h}}-k_{2}a-k_{3}ba,$$

$$\frac{db}{dt}=k_{4}(\bar{a}-k_{ss})b$$ (1)

$$\bar{a}=(\oint_{\Gamma }ads)/\left(\oint_{\Gamma }1ds\right),\;p=\frac{1}{1+(\beta u{)}^{-q}}.$$

The novelty of this work is that we solve this system of equations on a growing surface in 3-dimensional space. Here, *a* represents the concentration of activated Cdc42 associated with cell surface *Γ*, and *b* represents the concentration of its negative regulators, such as the protein kinase Cla4p which is activated by Cdc42 and inhibits it [65]. $\oint_{\Gamma }*ds$ denotes the integral over the surface of cell membrane *Γ*, and Δ_*Γ*_ denotes the Laplace-Beltrami operator on *Γ*. An initial biased spatial cue *u*, for example, the pheromone gradient in extracellular space, was introduced to determine the location of the polarization site. More details of each individual term can be found in the Supplemental Information in (38). In particular, *u* is varied in time to represent different levels of polarization at different stages of one cell cycle. The maximal value of *u* was set to be achieved at the tip of the bud, whereas its minimum was set to be achieved at the point on the surface of the mother cell directly opposite to the bud site. The gradient *u*(*t*) is modified to be ${u(t)=u}_{min}+(u_{max}-u_{min})\cdot ((H_{T}-H_{min})/H_{total}{)}^{n(t)}$, where *n*(*t*) is the scaling factor such that higher values give rise to a steeper transition between the maximum and minimum of *u*. In particular, *n*(*t*) is non-static, and increases at the early stage followed by a reduction to generate a time-varying polarization, instead of a fixed constant.

The numerical scheme for solving Eq (1) is described in detail at the end of theS1 Text. By simulating the signaling reaction-diffusion submodel over the bud surface provided by the mechanical model, a spatially based distribution of Cdc42 is established and maintained on the cell surface (Fig A(A) in S1 Text). The level of polarization, including the size of the polarization site, is determined by the sharpness of the initial cue *u* and self-enhancement. At the same time, the solution of the reaction-diffusion equation is used to determine the location for cell surface materials insertion, leading to the growth of the bud surface. Namely, when the solution on the modeled cell surface patch exceeds a threshold value, such patch becomes eligible for cell surface expansion by adding new triangles into the existing cell surface mesh. (For a detailed description of the cell surface expansion method please see [43]). The robustness of the reaction-diffusion signaling submodel is tested on a cell surface mesh that undergoes Monte Carlo remeshing periodically. The results showed that the numerical scheme is stable. Furthermore, the stability is maintained when applied to a dynamical cell surface mesh where both the Monte Carlo remeshing and cell surface expansion are introduced (Figs 3 & 5).

## Supporting information

S1 Text Methods for image analysis and model setup and calibration details. (DOCX)

S1 Video Simulation video corresponding to snapshot shown in Fig 3A for spherical budding. (AVI)

S2 Video Simulation video corresponding to snapshot shown in Fig 3B for tubular budding. (AVI)
