# Supplementary material for: Study of impacts of two types of cellular aging on the yeast bud morphogenesis
Source: PLoS Comput Biol. 2024 Sep 30;20(9):e1012491. doi: 10.1371/journal.pcbi.1012491 (PMC11476777; doi:10.1371/journal.pcbi.1012491)
Supplement: S1 Text — (DOCX) [file pcbi.1012491.s001.docx]

## **Supplementary Information for**

Study of Impacts of Two Types of Cellular Aging on the Yeast Bud Morphogenesis

##

Kevin Tsai^1,2^ , Zhen Zhou^4^, Jiadong Yang^5^, Zhiliang Xu^9^, Shixin Xu^8^, Roya Zandi^2,3,7^, Nan Hao^4^, Weitao Chen^1,2,5,7,∗^ and Mark Alber^1,2,6,7,∗^.

1 Department of Mathematics, University of California, Riverside, CA, United States of America

2 Interdisciplinary Center for Quantitative Modeling in Biology, University of California, Riverside, CA, United States of America

3 Department of Physics and Astronomy, University of California, Riverside, CA, United States of America

4 Department [of Molecular Biology, School of Biological Sciences,](http://biology.ucsd.edu/research/academic-sections/mb/) University of California,San Diego, CA, United States of America

5 Department of Molecular, Cell and Systems Biology, University of California, Riverside, CA, United States of America

6 Department of Bioengineering, University of California, Riverside, CA, United States of America

7 Biophysics Graduate Program, University of California, Riverside, CA, United States of America

8 Duke Kunshan University, Kunshan, Jiangsu, China

9 Applied and Computational Mathematics and Statistics Department, University of Notre Dame, Notre Dame, IN, United States of America

##

**Corresponding authors:** Mark Alber, Weitao Chen.

**Email:**  [malber@ucr.edu](mailto:malber@ucr.edu), weitaoc@ucr.edu

## **Imaging.** Time-lapse images were taken by using a Nikon Ti-E inverted fluorescence microscope equipped with an EMCCD camera (Andor iXon X3 DU897) and a CFI plan Apochromat Lambda DM 60X oil immersion objective (NA 1.40 WD 0.13MM). The microfluidic device loaded with yeast cells was fitted onto the motorized stage upon objective. Images were acquired every 15 min for a total of 90 hours or more with the exposure setting of 50 ms for the phase channel. The detailed method has been described previously [(1–4)](https://www.zotero.org/google-docs/?Zu1cKB).

## **Image analysis.** Image analysis was performed using the image processing package Fiji (https://imagej.net/software/fiji/). Samples were selected from time-lapse data according to the cell cycle time and cell geometry for each individual bud. The aspect ratio extracted from the experimental images was calculated using the long and short axes of the cells of interest via manual measurement. The measurement was performed using the “straight” line function in Fiji to measure the longest and shortest axes, approximately mutually orthogonal, of each bud. In addition, the width of the mother-bud junction is measured to approximate the width of the septin and chitin ring structure. Such width was then used to calculate the approximate initial bud surface area. We identified the initiation of a bud by one video frame (15 minutes per frame) before the first visual observation of a visible protrusion on the cell surface. Due to the 2D nature of the image, we assumed that the mother cells and buds attain axial symmetry along the axis parallel to the mother–bud orientation and the shape is equivalent to that of an ellipsoid. Thus, the bud surface and volume were approximated using the ellipsoidal surface area and volume formula, respectively. Young cell data was collected at the first possible generation in each data set. Old cell data was collected at the fourth generation before cell death for the same mother cell.

## **Model setup.** In the computational model, the mother cell is represented as a coarse-grained 2D triangulated surface, embedded in 3D space, with dynamical re-meshing and growth capability. Re-meshing, local membrane deformation, and bud growth are carried out following a Markov chain process. The chemical signaling submodel developed in this paper is based on the computational implementation of a system of reaction-diffusion equations on the growing cell membrane in 3D space. Moreover, cell polarization is obtained in the model due to a biased spatial gradient of some extracellular chemical substance of interest. For a detailed description of the chemical signaling model, please see [(5)](https://www.zotero.org/google-docs/?Oisx3d). The reaction-diffusion equation describing a chemical signaling network is numerically solved on the triangular mesh on the growing cell surface obtained in the mechanical model with re-meshing and mechanical relaxation. A typical simulation has the reaction-diffusion equation initiated and solved for every 25 re-meshing-then-mechanically-relaxed operations. When numerically solving the reaction-diffusion equation, a total of 5000 iterative steps are involved each time to reach the quasi-steady state.

**Mechanical submodel.** The mechanical properties of the model surface are represented by appropriate choices of energy potential describing an elastic material. The movement of each node of the triangulated surface is governed by the force acting on it. This force is calculated via the negative gradient of the corresponding energy potentials. For detailed model descriptions regarding the choice of energy potential, re-meshing algorithm, and growth algorithm, please refer to [(6)](https://www.zotero.org/google-docs/?636jnR).

**Coupled mechano-chemical model.** The coupling of the mechano-chemical model is established via the mutual feedback performed by allowing a cell membrane to evolve for a given time period and afterward, allowing the chemical concentration to be updated on the new 2D surface in a 3D space. Spatial information such as the global x,y, and z-coordinates and local surface curvature are utilized in the chemical signaling model, a system of reaction-diffusion equations, to determine the change in local chemical concentration. A conversion from global coordinates into curvilinear coordinates enables the proper treatment of the Laplace-Beltrami operator in the reaction-diffusion equations. Note that the chemical signals achieve steady state faster than cell morphological changes. The mechanical model utilizes the local chemical concentration obtained as a quasi-steady state from the chemical signaling model to determine the local mechanical properties and/or the suitable location for new cell surface material insertion that drives the growth of a cell. Therefore, such coupling is multiscale in both time and space.

**Model calibration.** To propose and test the necessary conditions for bud emergence, we have used a newly developed 3-dimensional discrete particle-based model, which takes into account anisotropicity in the cell surface mechanical properties and dynamical re-meshing of the model geometry. We proposed that the cell surface at the budding site must undergo weakening (or softening), particularly in the resistance to bending deformation, for a bud to emerge. In this work, we calibrated the model by performing a systematic study on model parameter perturbation to observe effects on the budding cycle duration and comparing the results with experimental data. Using the computational model, we first investigated how the mechanical properties of the bud affected the bud growth trajectory to identify possible changes in mechanical properties leading to different cell cycle lengths for spherical budding. In particular, we perturbed the cell surface material insertion period ($N\Delta t$), stretching stiffness ($k_{s}$), bending stiffness ($k_{b}$) of the bud surface, and the threshold value on the surface expansion ($\gamma$) required to allow new material insertion to simulate single cell budding. For each simulation, we quantified the bud surface area throughout the budding and plotted the growth trajectory in time, as shown in Fig A. Notice that, there was no mechanism for the termination of the budding process incorporated in the model. Therefore, when the bud-to-mother cell surface area ratio was similar to the experimental data, the simulations were stopped. We perturbed those critical parameters and studied their effect on the budding initiation and the linear growth rate of the budding trajectory.

We first noticed that, within the parameter regime such that a bud could occur, homogeneous mechanical properties and constant new material insertion rate within the bud region always gave rise to a linearly bud growth. In addition, the bud shape obtained was always spherical in simulations. By perturbing the cell surface material insertion period, i.e., the number of time steps between successive growth events $N$, a shorter insertion period had the bud growth initiated earlier. As studied in our previous work [(6)](https://www.zotero.org/google-docs/?VkX2pk), bud emergence was determined based on the visibility of a protrusion from the cell surface with a volume of at least 5.5% of the total cell volume. Simulation results showed that once the bud started to grow, the bud growth rate was dependent on the new material insertion period, i.e., smaller $N$ gave rise to a larger budding growth rate (Fig A(A)).

Changing the mechanical properties such as stretching and bending stiffness only influenced the budding initiation time without affecting the growth rate (Fig A(B)). Reducing the weakening of the bending stiffness or enhancing the weakening of the stretching stiffness within the bud surface initiated the budding earlier, without changing the bud growth rate afterwards. This was surprising since mechanical properties should affect the bud surface area growth. The spatially homogeneous material insertion under different mechanical properties might have led to a similar growth rate due to the sufficient relaxation implemented between successive growth events. Perturbing the threshold value for surface expansion ($\gamma$) affected the bud initiation mostly, but only a marginal effect was observed on the budding growth rate after the initiation (Fig A(C)). Larger $\gamma$ led to a longer time to start the bud growth. More specifically, as shown in Fig A, when $\gamma=0.025$, all simulated samples started fast budding simultaneously, which was about 5 min after the cell division in the previous cell cycle. For $\gamma=0.075$, the average initiation time was about 20 min. When $\gamma=0.1$, the bud did not grow significantly until 40 min after the cell division in the previous cell cycle. The growth rate after the bud was initiated was not affected by $\gamma$ too much, i.e., the budding growth trajectory exhibited a similar growth rate for $\gamma=0.025, 0.075,$ and $1$. The variation in the growth trajectory among different simulated samples using the same parameter set was mainly due to the initiation time. The bud size from the simulations also remained similar for different $\gamma$ (Table A). Therefore, the threshold value for surface expansion in the algorithm affected the bud initiation significantly without changing the growth rate.

| 1. 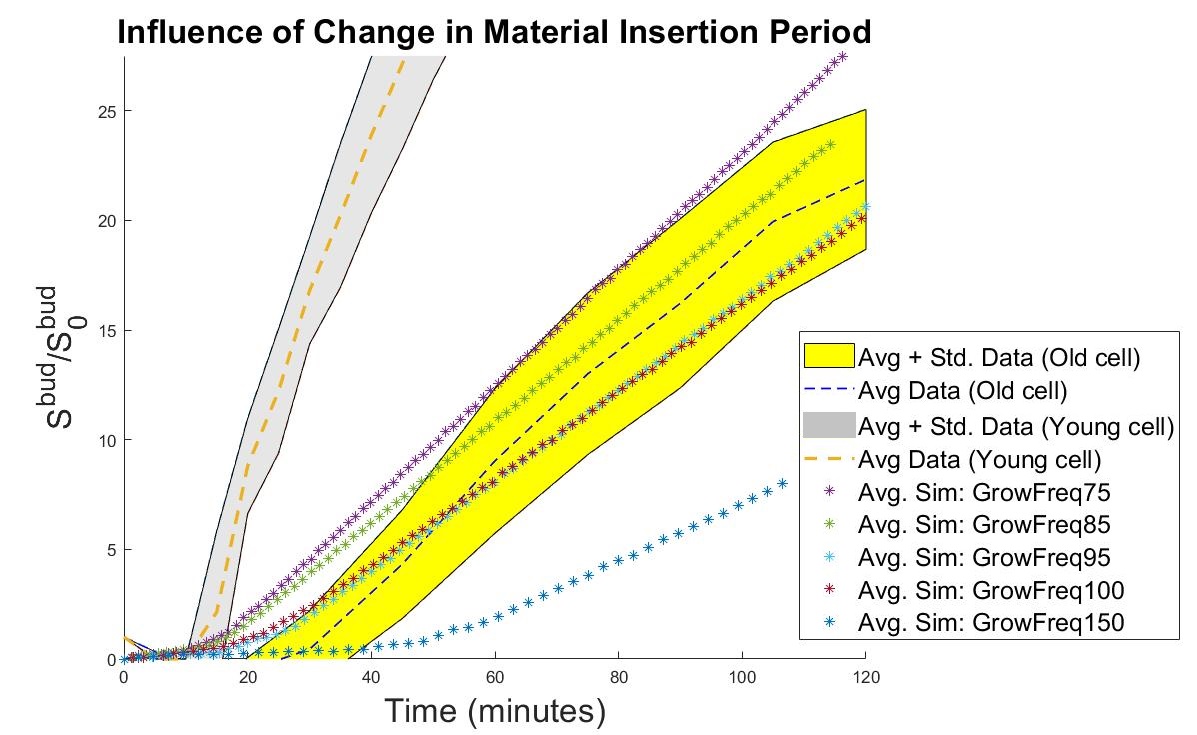 2. 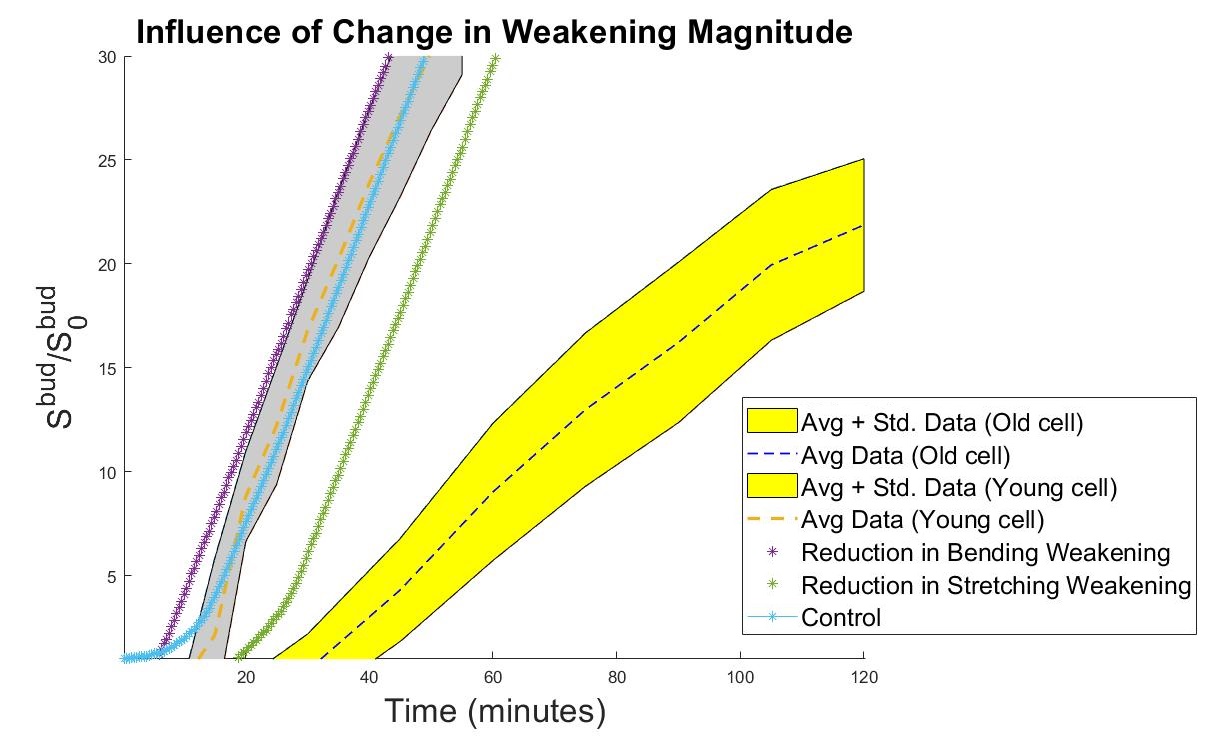   (C) 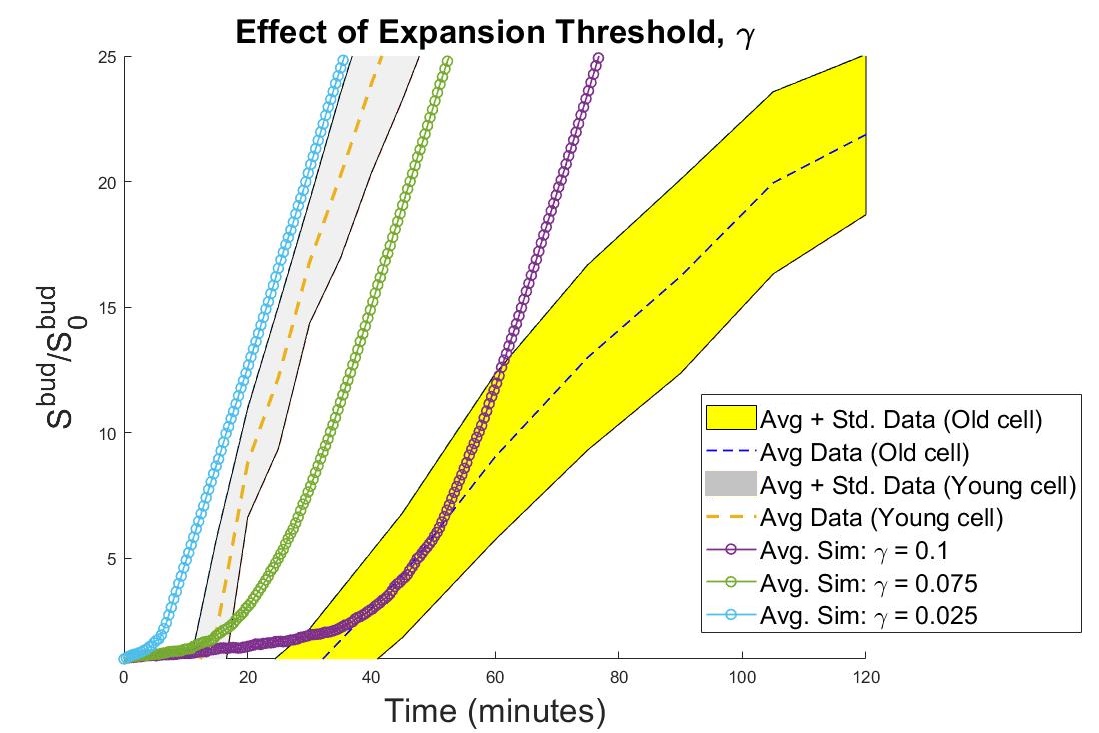 |
| --- |
| **Figure A.** Possible changes in Mode 2 with spherical budding during cellular aging. Effect of changes in cell surface material insertion period (A) and changes in the stretching or bending stiffness (B). The y-axis represents the ratio of the bud surface area with respect to the estimated initial bud site area enclosed by the septin and chitin ring. Experimental data is collected from aged cells. (C) Effect of changes in the threshold value of surface expansion ($\gamma$). Increasing $\gamma$ led to delayed bud initiation. Bud growth trajectories showed marginal differences after bud initiation. Data presented here is extracted from young cell data. |

The simulation results suggested that, for spherical budding, the growth rate was only affected by the cell surface material insertion period, whereas the bud initiation time could be affected by the mechanical properties, the new material insertion rate, or the cell surface expansion threshold within the bud region. This indicated that the new cell surface materials were delivered less frequently to the bud region as cells aged in Mode 2. The delay of the bud initiation could be due to the less frequent new cell surface material insertion, enhanced weakening of the mechanical properties, or a larger allocated space required for new material insertion. Therefore, appropriate parameter values were chosen in the model such that similar bud initiation time and growth rate as the experimental data could be obtained (Fig B).

| 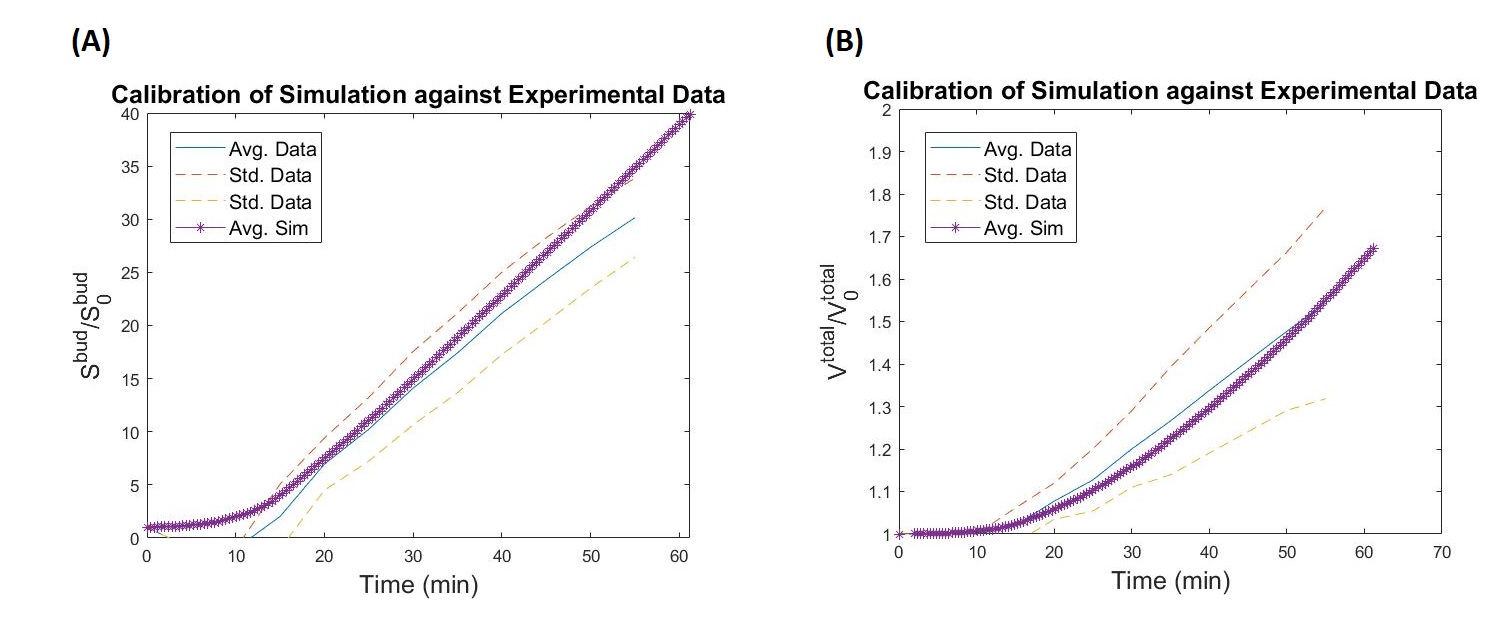 |
| --- |
| **Figure B.** Calibration against the experimentally observed budding cycle duration for young yeast cells. (A) Ratio of bud surface area with respect to initial bud surface area, and (B) Total cell volume with respect to initial total cell volume. 10 simulations were performed to ensure statistical significance. The average simulation trajectory is compared to the observed data range. Observed data range is presented as the mean trajectories with standard deviations. |

More specifically, 10 simulations were performed for each parameter set during calibration for statistical significance. Our calibration results suggest that using the parameter set $(\alpha_{s}, \alpha_{a}, \alpha_{b}) = (0.75, 0.135, 0.75)$ coupled with the surface area expansion threshold $\gamma=0.05$ and a single cell surface expansion event accepted every $1250\Delta t$ unit time, where $\Delta t = 8\times10^{-5} min$ is the simulation time step size, nine out of ten simulations results fell close to or within the experimentally observed bud cycle duration for the young wild type yeast cell. Even though this choice of parameter set produced results in close agreement with the experimental data, this does not imply that there are no other working parameter sets due to a lack of precise time-series data on the mechanical properties of budding yeast cells.

**Stability analysis of the mechano-chemical model.** In order to solve differential equations involved in the chemical signaling submodel on the cell surface obtained in the mechanical submodel, we adopted the locally discontinuous Galerkin method. This method utilized the local coordinate transformation and local curvature information, which can effectively capture the effect of surface curvature on the diffusion operator, and is ideal to handle the Laplace-Beltrami operator in the 3D setting. However, since our mechanical model incorporates stochastic components that can alter the connectivities between surface nodes in the mesh, it is essential to first investigate the stability of the numerical method on such a dynamical mesh. To evaluate the stability, we fixed a parameter set and solved the differential equations on a spherical surface with multiple trials. The results from multiple trials suggested that the method is stable without any sign of numerical “blow-ups” or similar problems (Fig C). It is worth noting that in this validation attempt, we did not require the polarization to converge to a steady state before triggering the growth algorithm since our interest here was to verify the stability.

| **A**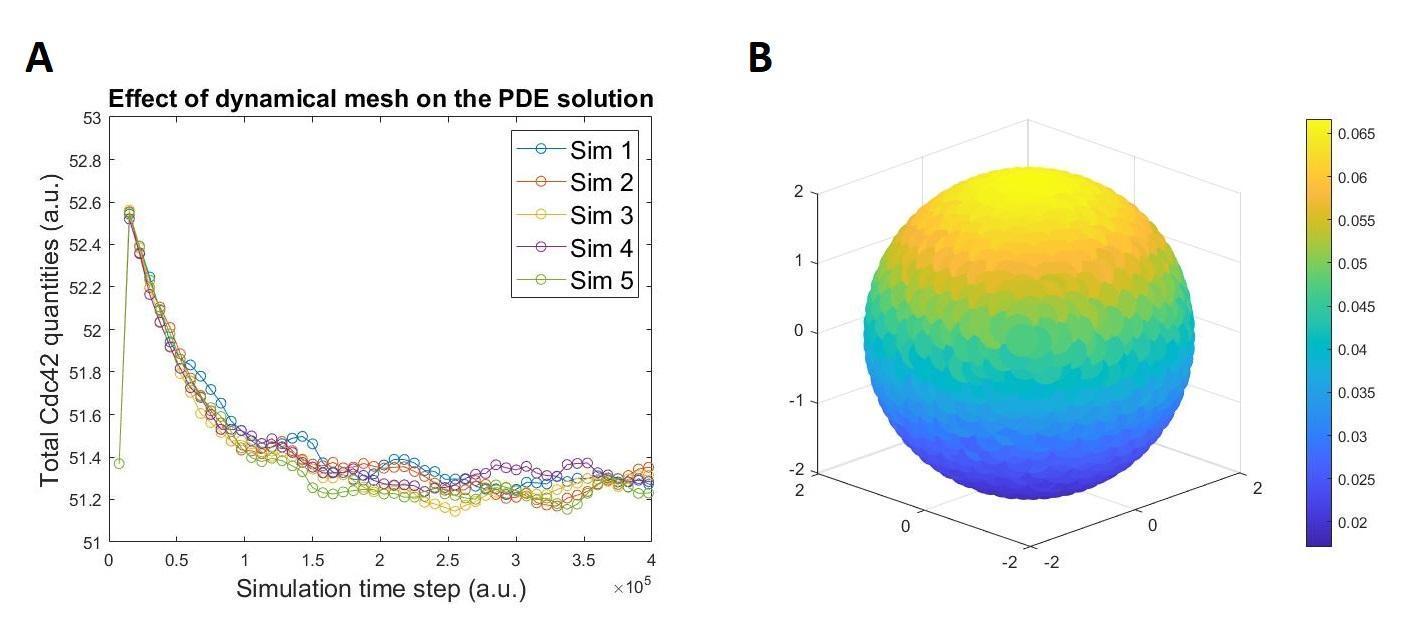**B**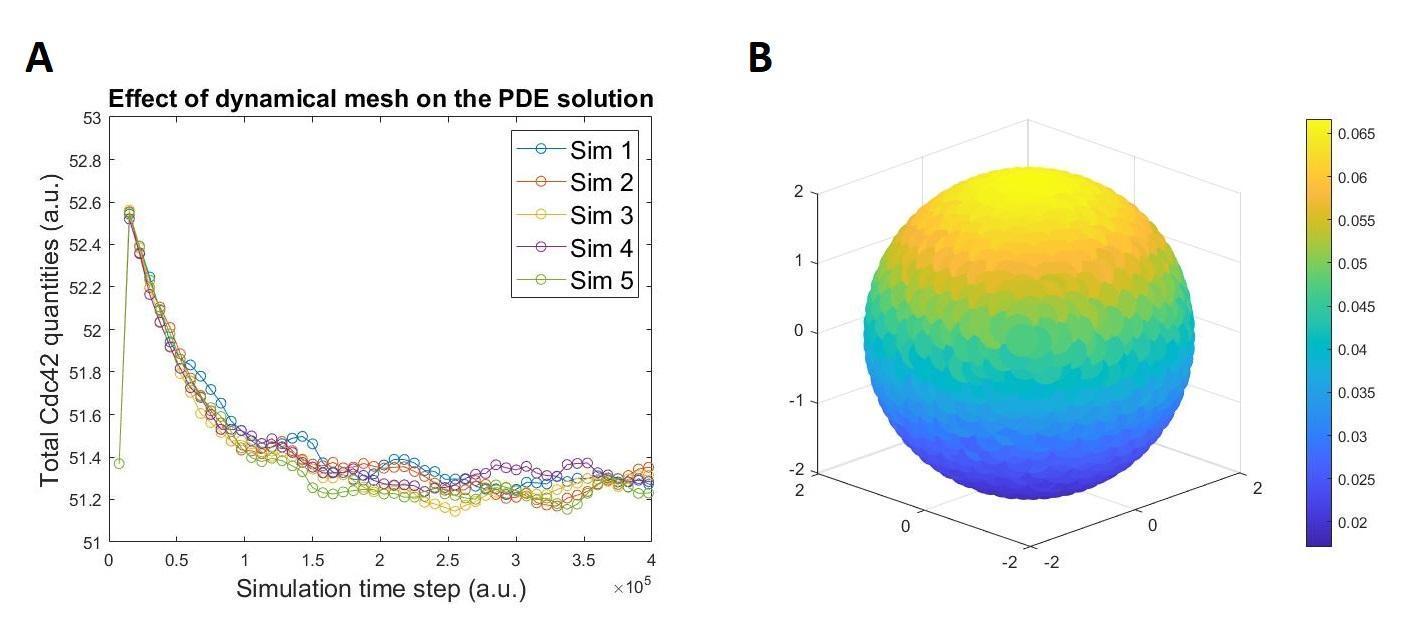**C**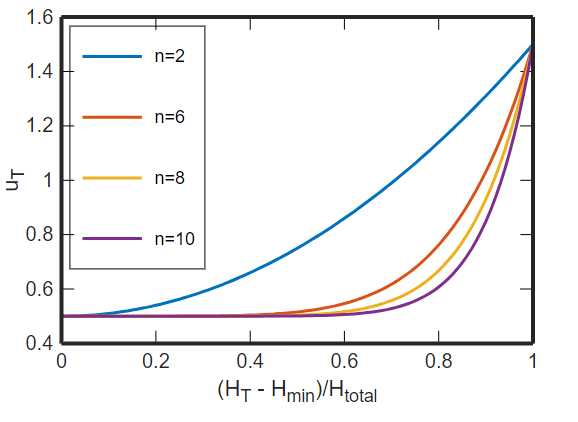 |
| --- |
| **Figure C**. (A) A simulated polarized distribution of active Cdc42 over the triangulated sphere. The size of the polarization zone can change depending on the input parameter used for solving the differential equations. (B) The total quantity (in arbitrary units) of the Cdc42 is shown to reach similar steady states in all sample simulations with the same parameter. The fluctuations are due to the dynamical mesh, a result from probabilistic Monte Carlo re-meshing, used in the simulation. (C) Profiles of the gradient $u{=u}_{min}+(u_{max}-u_{min})\cdot((H_{T}-H_{min})/H_{total})^{n}$, which serves as the spatial cue of Cdc42 polarization, with different sharpness represented by parameter $n$. |

To evaluate the effect of biased cell surface material insertion, we changed the parameter used in the PDE to emulate different chemical distributions to govern the location of cell surface material insertion. We first fixed the following parameters in the chemical signal model: $k_{0}=20, k_{1}=25, k_{2}=k_{3}=5, k_{4}=1, k_{ss}=12,\gamma=1, q=h=10$. In addition, $u$, which controls the bias in production of $a$ is set to be $u_{max}=1.5$ and $u_{min}=0.5$. Instead of the linear interpolation between $u_{max}$ and $u_{min}$ used in [(5)](https://www.zotero.org/google-docs/?zcGzGY), the interpolation is made non-linear such that $u = u_{min} + L_{i}(u_{max}-u_{min})$ where $L_{i}=(|z_{i}-z_{tip}{|/|z_{min}-z_{tip}|)}^{4}$ and $z, z_{tip}, z_{min}$ are the z-coordinate of the center of $i$th triangle, and z-coordinate of the tip and bottom-most point, respectively. In addition, we assumed that only a location with a local chemical concentration exceeding $0.8a_{max}$($a_{max}$ is the global maximum chemical concentration) is eligible for material insertion.

| 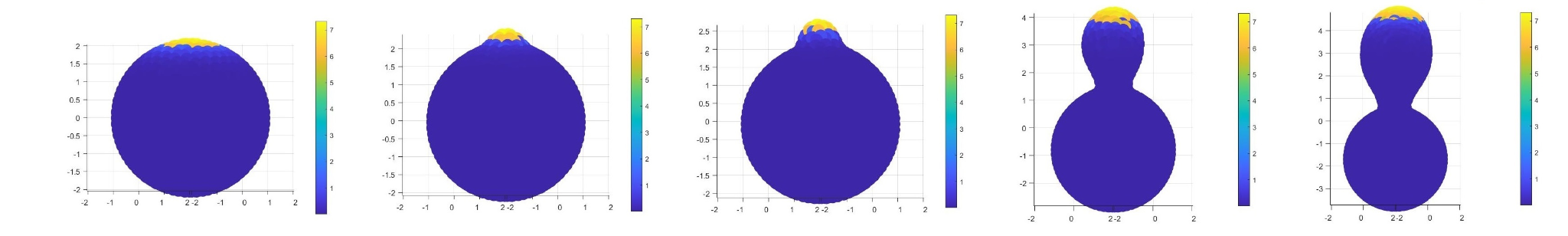  **A**  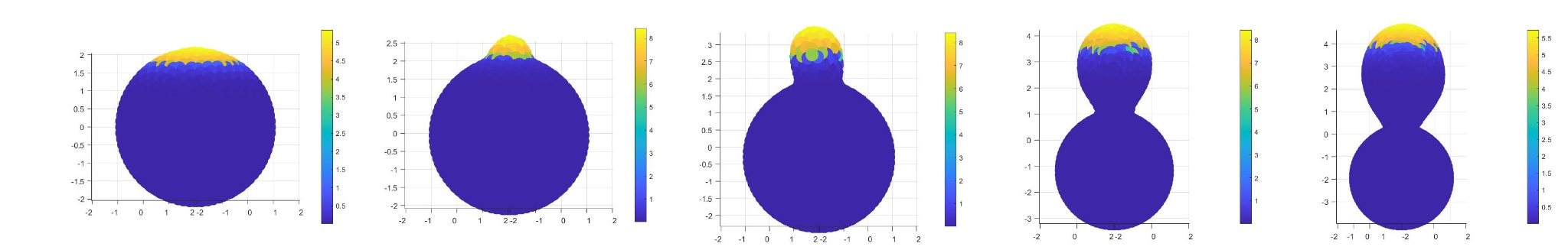  **B**  **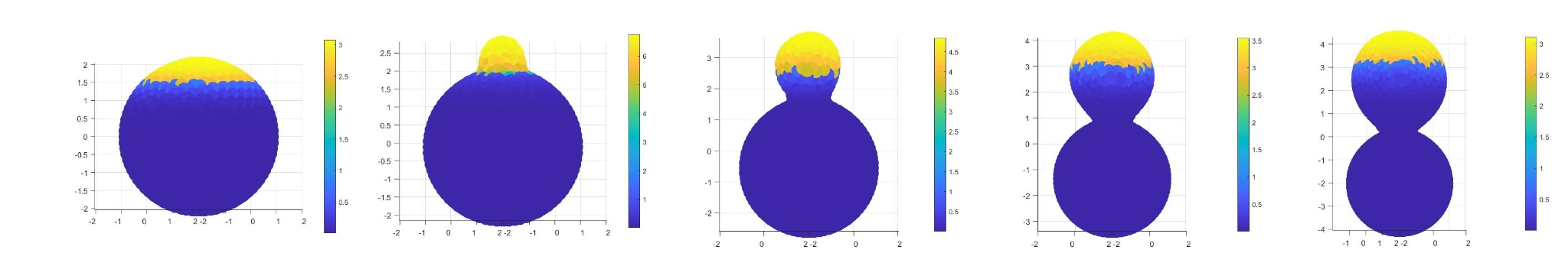**  **C** |
| --- |
| **Figure D.** (A) $\beta=0.489$, (B) $\beta= 0.8$, (C) $\beta= 1.0$. Time lapse is oriented left to right with left-most images depicting the initial polarization profiles and the right-most images depicting the final bud shape. The brightness of the color indicates the local chemical concentration effectively determining the location of material insertions. Brighter color indicates the region eligible for material insertions. |

$\beta$ is varied between $\beta=0.489, 0.8, 1.0$ to change the size of the polarization zone such that $\beta=1.0$ leading to the largest polarization zone (Fig D). Based on this setup, we observe different bud shapes transitioning from a relatively narrow ellipsoid to a sphere, corresponding to the increased size of the region eligible for cell surface material insertion. This suggests that the bud shape depends on where new material can be inserted, and elongation requires a highly restricted zone of material insertion or a highly polarized chemical distribution responsible for directing new materials.

**Tubular budding cannot be generated by nonuniform mechanical properties over the bud surface within the regime of parameters required for bud initiation.** It had been shown that homogeneous mechanical properties over the bud surface could only generate a spherical bud shape [(6)](https://www.zotero.org/google-docs/?lOb8io). Therefore, we tested whether the anisotropic mechanical properties of the bud surface were sufficient to give rise to a tubular bud shape. In our previous study [(6)](https://www.zotero.org/google-docs/?tMUZxs), we used a Hill function to model the anisotropic mechanical properties and tested a case that the anisotropicity occurred before the bud formation, based on the assumption that the cell polarization failed to be established uniformly within the bud region, and found that nonbiological bud shapes could occur. More specifically, a “bud neck” was formed away from the septin and chitin ring position. In this study, we also used the Hill function to model the spatially varying mechanical properties over the bud surface with additional new assumptions. We assumed that the anisotropicity did not occur until a bud was initiated, i.e., during bud initiation, the bud surface had uniform mechanical properties. Furthermore, the midpoint between the maximally weakened mechanical properties of the bud surface and the mother cell surface was placed either at the septin and chitin ring position or halfway between the bud tip and the septin and chitin ring position. These new assumptions were made based on the observation that the distribution of the polarized signal which governed the budding process was uniform over the bud surface during bud initiation, but became nonuniform after the formation of a small bud [(7)](https://www.zotero.org/google-docs/?IGIZ4j).

| 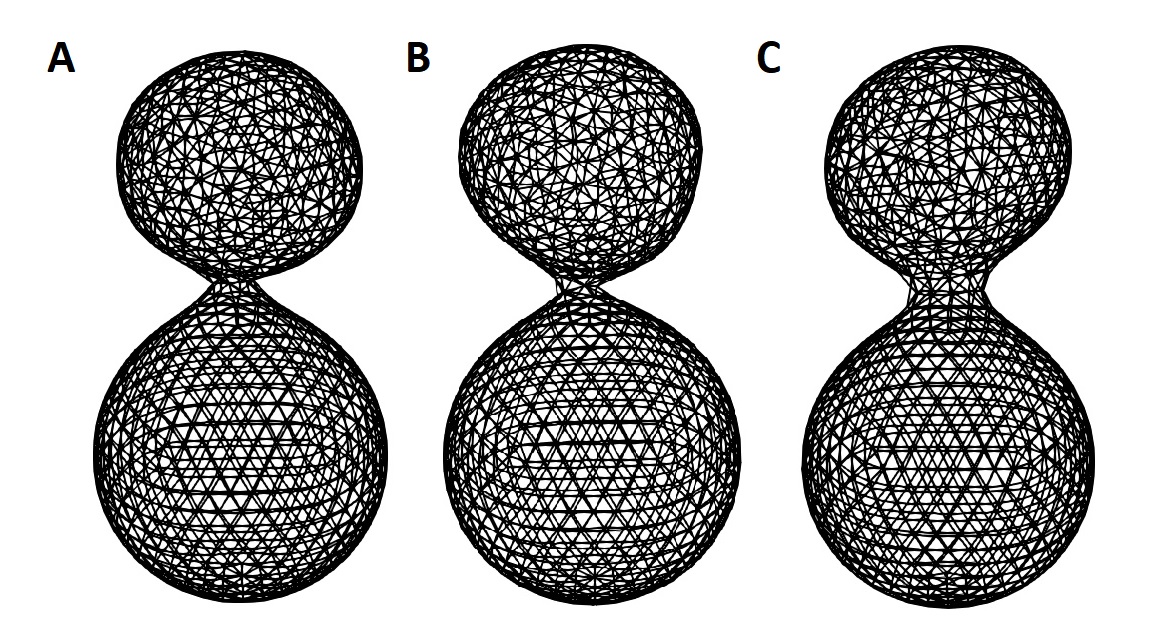 |
| --- |
| **Figure E.** Buds with narrow necks, similar to results obtained when the Hill function of $u$ had a relatively sharp transition ($n=16$). (A) Introducing the anisotropic mechanical properties from the beginning. (B) Introducing the anisotropic mechanical properties when $DR=4R_{min}$. (C) Introducing the anisotropic mechanical properties when $DR=6R_{min}$. The bud neck was more narrow if anisotropic mechanical properties were introduced earlier. Images were captured at the time point when the bud volume is about $50\%$ of the mother cell. |

To satisfy that the midpoint was at the septin and chitin ring position in the first case, the parameter sets were chosen to be (1) $(k_{s}, k_{a}) = (1.0, 1.0)$ and $k_{b}$ was varied between 0.18 and 1.0 spatially satisfying that $k_{b}=0.18$ at the tip of the bud (and also uniformly at the budding site during the bud initiation) and was increased as moving toward the septin and chitin ring location, (2) $k_{b} = 0.135$ and $k_{s}, k_{a}$ was varied between 0.5 and 1.0 spatially satisfying that $k_{s}, k_{a}=0.5$ at the tip of the bud. The simulation results with high Hill coefficients still generated a “bud neck” formed away from the septin and chitin position due to the sharp change in the mechanical properties (Fig E), which was consistent with the results obtained in our previous study [(6)](https://www.zotero.org/google-docs/?95aoCX). Such bud formation was not sensitive to the timing of introducing the anisotropic mechanical properties which was characterized by the height of the bud obtained at that moment (termed delayed repolarization, $DR$, in our model) (Fig E). Shapes of buds generated in this case remained more or less spherical. In the second case such that the midpoint of the Hill function was located halfway between the bud tip and the septin and chitin ring position, a spherical bud similar to the experimental observation was always obtained with different Hill coefficients and different timing of introducing the anisotropic mechanical properties (Fig F, and Table D, E). Therefore, the anisotropic mechanical properties along the bud surface failed to produce tubular budding.

| 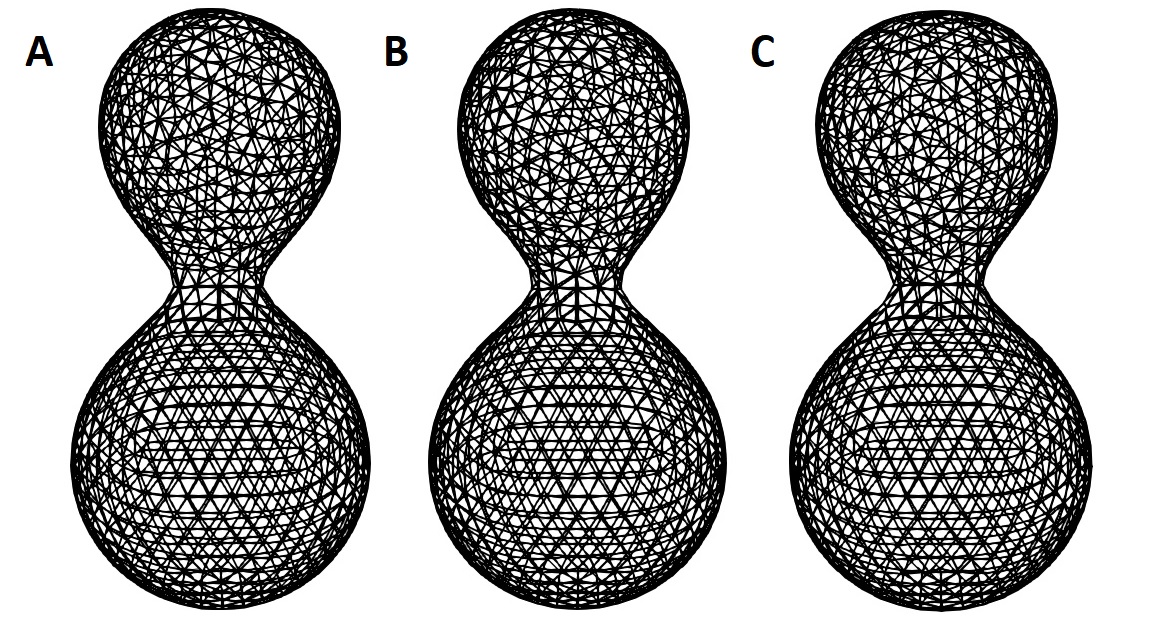 |
| --- |
| **Figure F.** (A) $n=8, K=0.5, DR=4R_{min}$. (B) $n=8, K=0.5, DR=6R_{min}$. (C) $n=16, K=0.5, DR=4R_{min}$. $K=0.5$ here indicated that the location where the Hill function of $u$ yielded a value equal to the middle of its minimum and maximum was at the midpoint between the tip of the bud and the averaged z-coordinate of the septin ring. Images were captured at the time point where the total cell volume is $1.5V_{0}$. The Hill function-type scaling is applied to the bending stiffness and stretching stiffness separately. |

**Perturbation of Growth Region.** We assumed that new cell surface materials could only be introduced within a subregion centered at the tip of the bud instead of the entire bud. This subregion was described by the Polarization Height (PH) in our model, defined as the height of this subregion from the tip of the bud (Fig G(A)). Such an assumption was made based on the fact that chemical signals that direct the budding process polarized in different regions within the bud at different times, followed by the growth-associated molecules, leading to a spatially non-homogeneous growth. Specifically, we tested $PH=2R_{min}$ corresponding to a relatively small subregion of growth and $PH=4R_{min}$ corresponding to a relatively large subregion of growth, where $R_{min}$ is chosen to be the equilibrium length of the linear spring potential used in our model for convenience. The simulation results showed that, for $PH=2R_{min}$, a tubular budding was generated (Fig G(A-B)). The aspect ratio of the bud shape kept increasing during the growth (Fig G(E)). The relative PH to the cell height was decreasing and always at low levels, except during the bud emergence (Fig G(F)). For $PH=4R_{min}$, a spherical bud was produced with an aspect ratio maintained around 1 (Fig G(C-D, G)). The relative PH to the cell height was also decreasing over time, but it was maintained at high levels at the early stage. Although at the late stage, it dropped to a similar level as the one observed for $PH=2R_{min}$, at the early stage (Fig G(H)), the spherical bud obtained was too large to change into a tubular shape. Together, these results suggested that the spatially biased growth (or cell surface expansion) alone was sufficient to give rise to tubular budding once the growth region relative to the cell size was sufficiently small at the early stage of budding, even with homogeneous mechanical properties over the entire bud surface. This could be due to the more restricted diffusion of the governing signaling molecules in Mode 1 due to cellular aging.

| **A**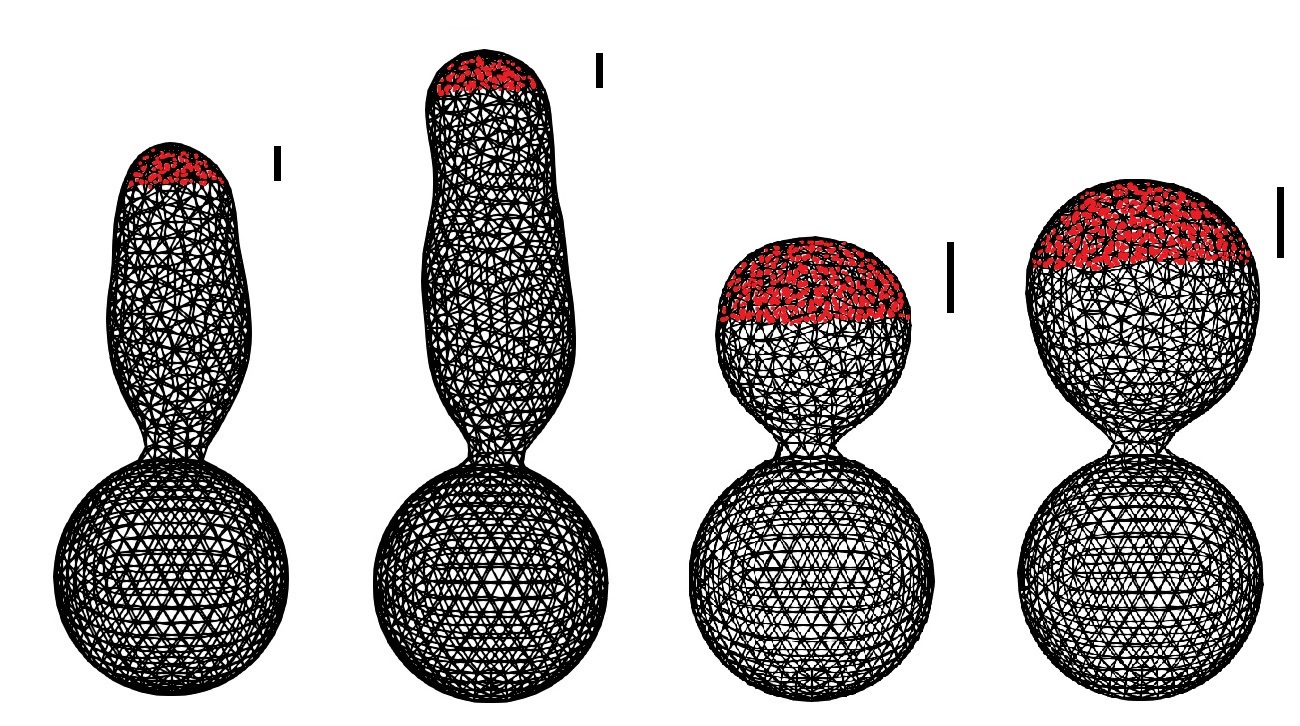**B 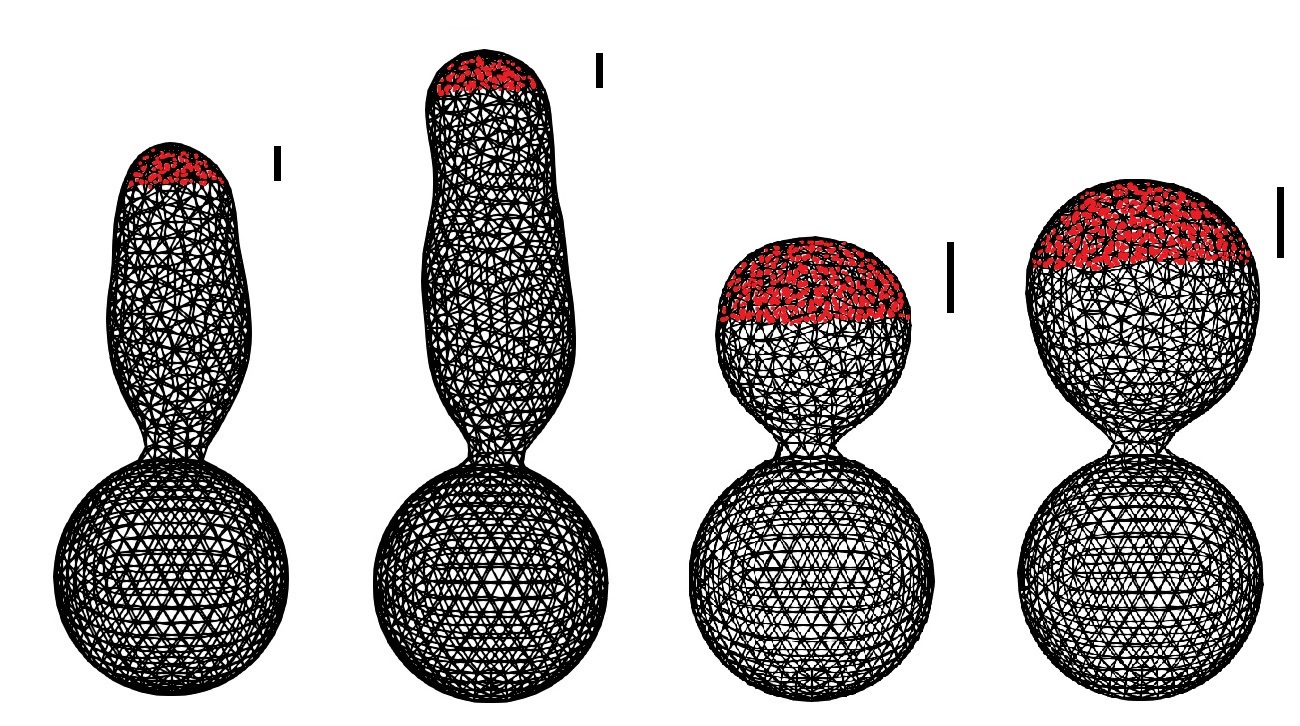C 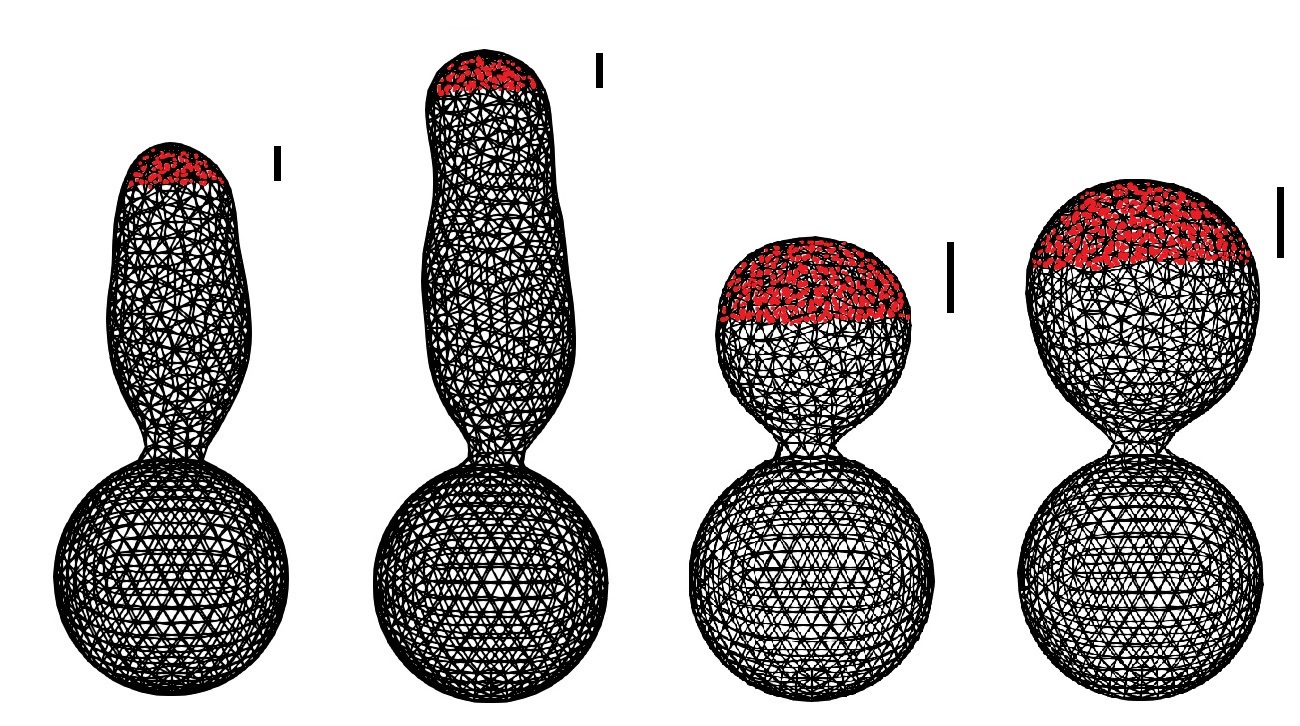D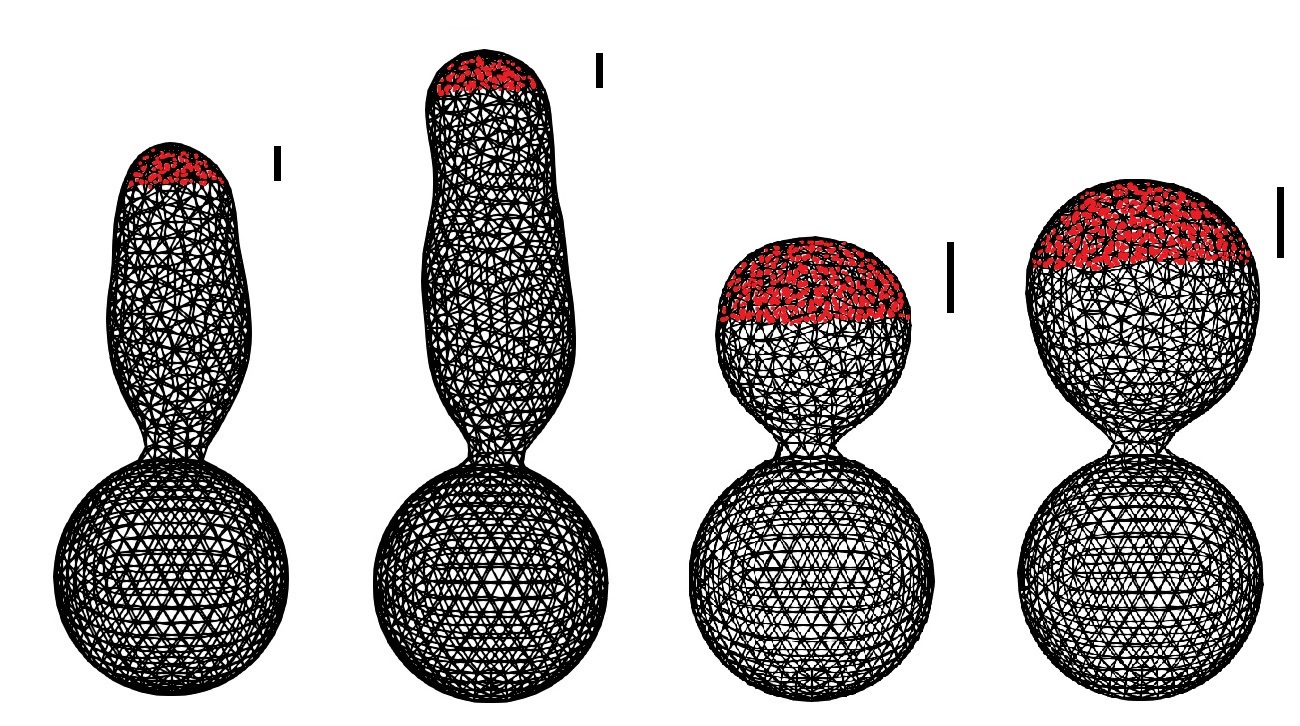**  **E 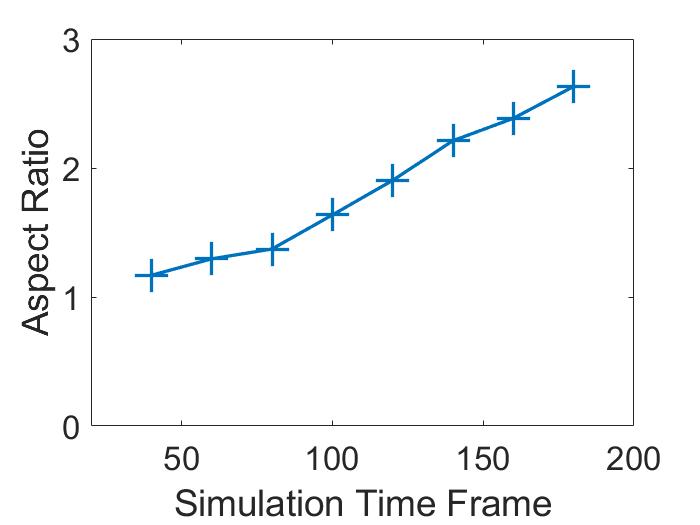 F 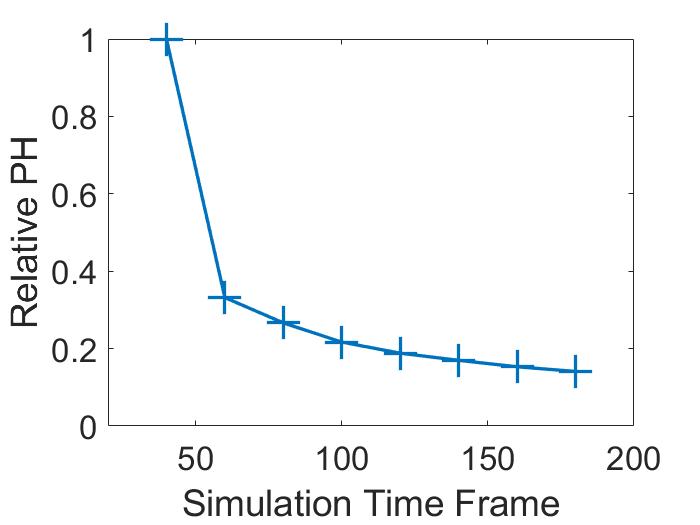**  **G 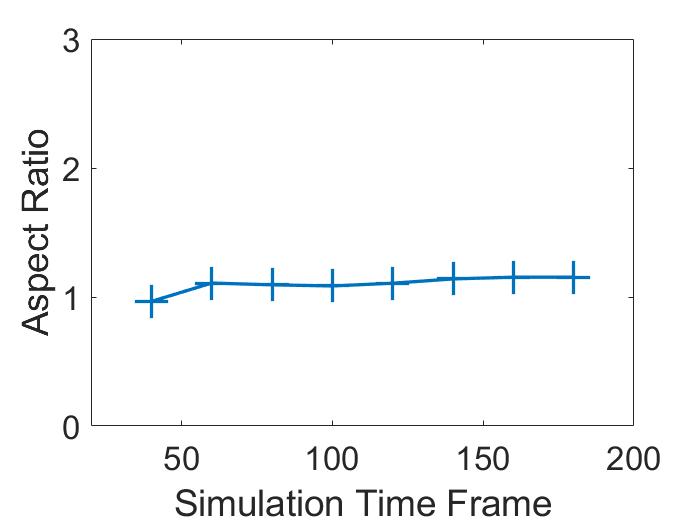H 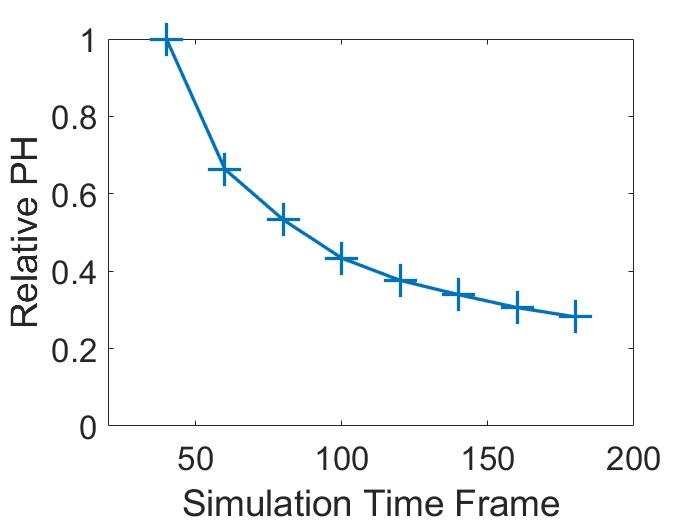** |
| --- |
| **Figure G.** Bud shape with spatially biased growth from the beginning of budding. (A) Cell volume of $1.5V_{0}$, $PH=2R_{min}$. (B) Cell volume of $1.76V_{0}$, $PH=2R_{min}$. (C) Cell volume of $1.5V_{0}$, $PH=4R_{min}$. (D) Cell volume of $1.97V_{0}$, $PH=4R_{min}$. (E) The averaged aspect ratio of the buds produced associated with the setup in (A,B). (F) The averaged relative polarized zone height (PH) with respect to bud height associated with the setup in (A,B). (G) The averaged aspect ratio of the buds produced associated with the setup in (C,D). (H) The averaged relative PH with respect to bud height associated with the setup in (C,D). Zones in red represent the PH where new materials are allowed to be inserted for growth. |

**Perturbation of Growth and Relaxation Regions.** During bud growth, enzymes involved in the cell wall modification actively cut and re-established connections among the cell wall components to allow growth and geometric change [(8)](https://www.zotero.org/google-docs/?broken=NgKdQo). However, the exact dynamics of the cell wall modification were yet to be fully understood, and it remained unclear whether the associated enzymes were concentrated near the growth tip or acted throughout the whole bud surface. To test the effect of the spatial range of cell wall modification governed by those enzymes, we altered the region where the re-meshing technique was applied in the simulation of tubular budding with the number of re-meshing steps fixed. When the re-meshing technique was only active at the locations where new cell surface materials were introduced, the tubular bud shape could be maintained during the growth to become extremely elongated (Fig H(A)). However, since the re-meshing was only restricted to the growing tip, the bud surface exhibited some undulation outside the re-meshing region. When the re-meshing was extended further away from the bud tip, the bud became shorter and wider with a less curved boundary (Fig H(B)). As we expanded the growth region, we were able to obtain a bud in a shape similar to the experimental results, which was wider at the tip and then shrunk toward the bud neck (Fig H(C-D)). However, the difference due to different re-meshing regions became less significant. Overall, these simulation results suggested that the region undergoing cell wall modification also affected the bud shape. Maintaining the tubular bud shape required less frequent cell wall modification in a restricted region within the bud surface.

| **A**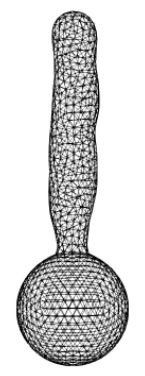**B**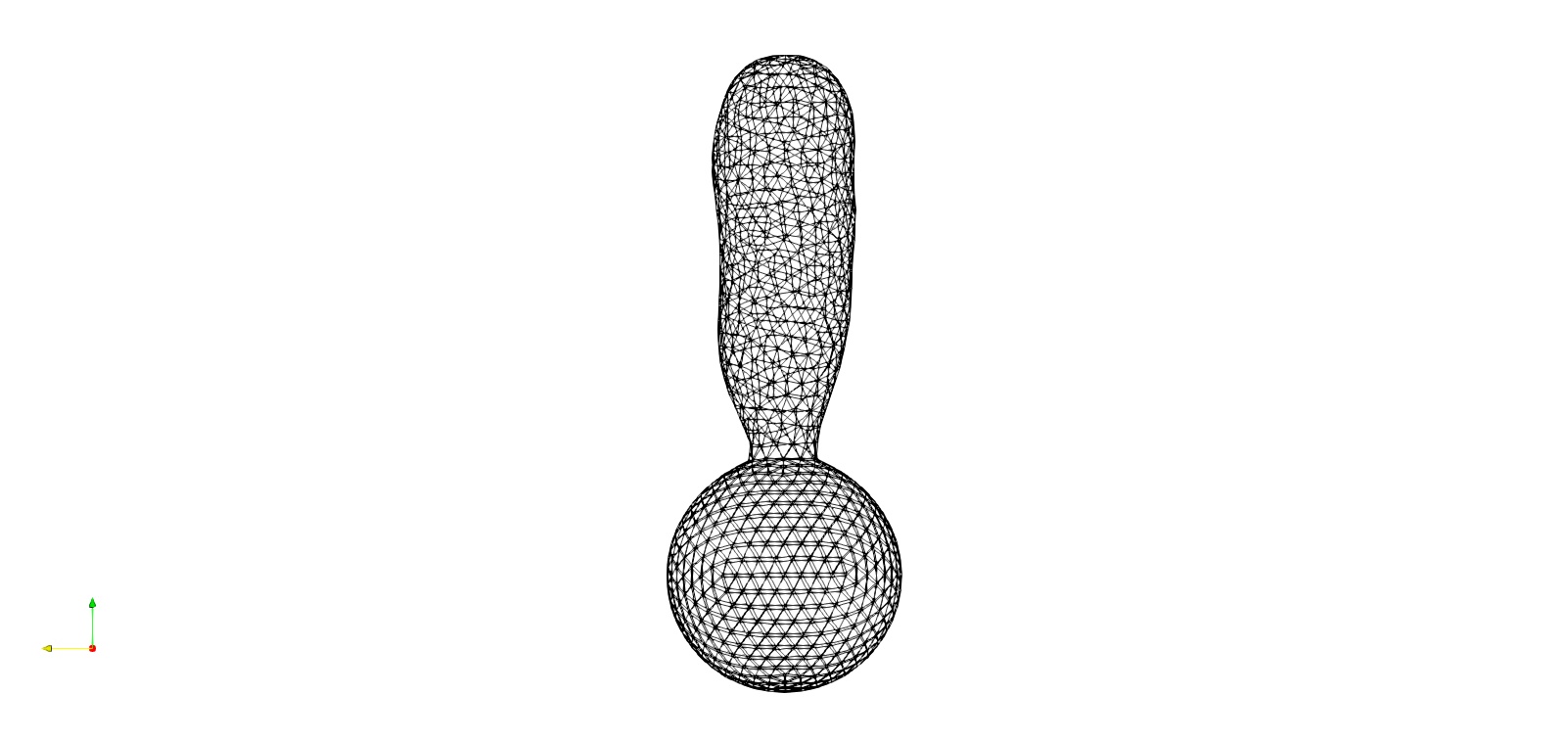**C** 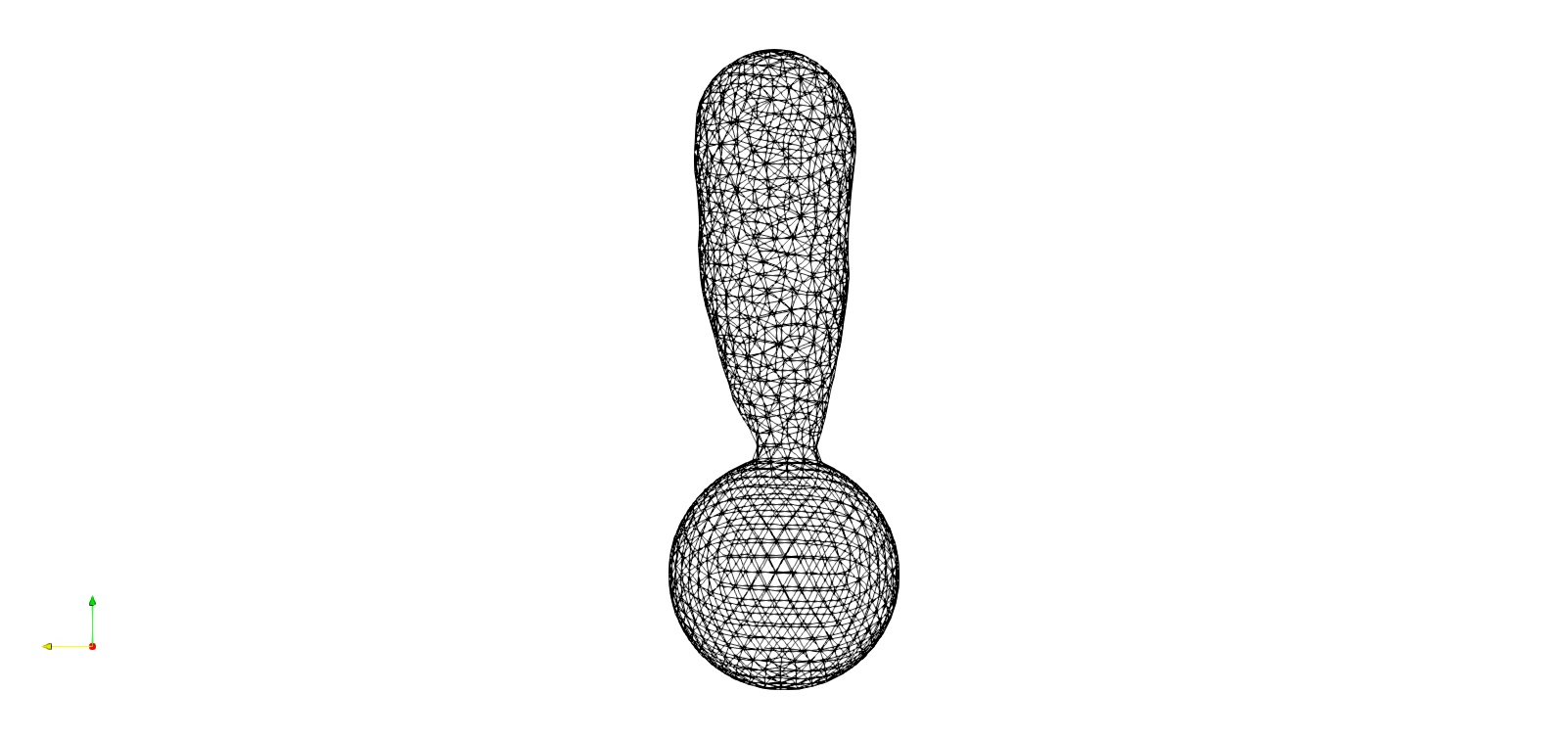**D** 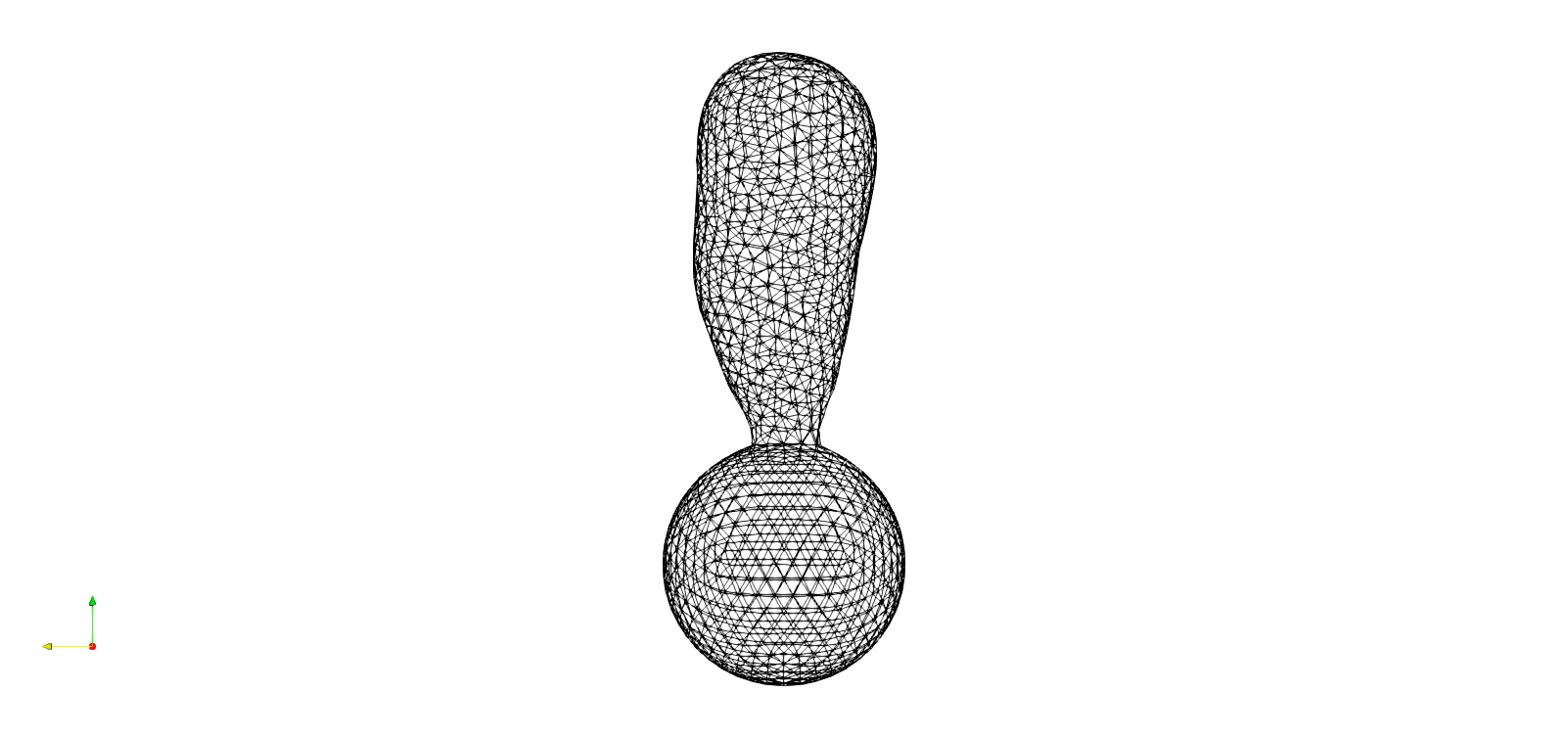 |
| --- |
| **Figure H**. Given a fixed height for material insertion, the size of the region undergoing re-meshing can influence the final bud shape. Re-meshing and growth are applied unbiasedly throughout the whole bud until the bud heights reaches $4R_{min}$. Afterwards, the re-meshing is restricted to regions whose height is within $2R_{min}$ from the bud tip (A), or restricted within $4R_{min}$ from the bud tip (B), whereas the material insertion is restricted within $2R_{min}$ from the bud tip. (C) Material insertion is restricted to $3R_{min}$ while the re-meshing is restricted to $2R_{min}$. Bud produced has an aspect ratio of 2.69. (D) Material insertion is restricted to $3R_{min}$ while the re-meshing is restricted to $4R_{min}$, and it produces a bud with a lower aspect ratio, 2.20, in comparison to (C) whereas the bud volumes are similar. |

**The transition from apically restricted growth to unbiased growth produces consistent bud shapes as experiments.** It has been described in earlier studies that the bud shape is determined by the timing of the transition from apical growth to isotropic growth [(9)](https://www.zotero.org/google-docs/?broken=AsyuxC). To test this phenomenon in our model, we first restricted the cell growth to areas that are less than $2L_{0}$, the distance (in height) away from the tip of the bud, where $L_{0}$ is the equilibrium edge length used in the model. The transition from apical growth to isotropic growth was set to occur when the height of the bud reached $10L_{0}, 14L_{0},$ and $18L_{0}$, respectively. Once the target bud height was reached, the cell was set to grow isotropically for a fixed amount of time which accounted for 40 additional frames in the visualization output. In agreement with known facts, different transition times determined the final bud shape, i.e., $10L_{0}, 14L_{0}, 18L_{0}$ led to the acquisition of a spherical, a short ellipsoidal, and a long ellipsoidal bud (Fig I).

| 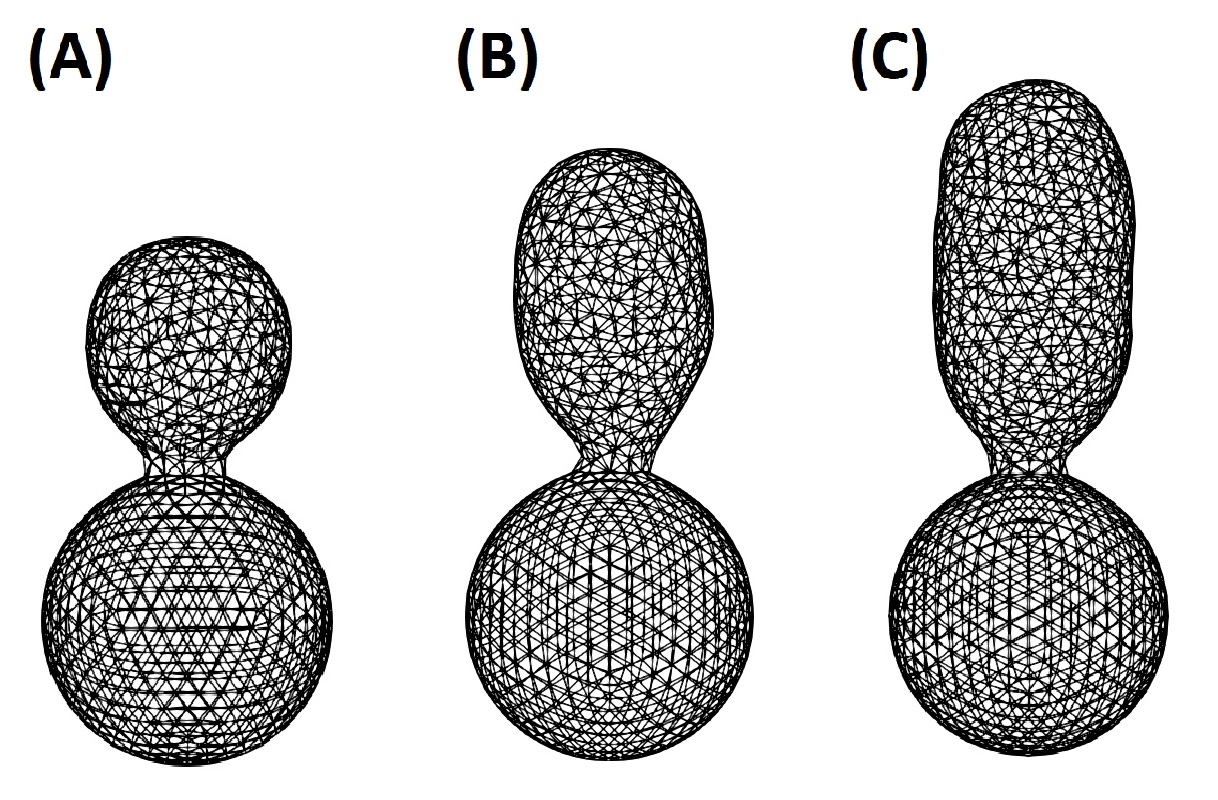 |
| --- |
| **Figure I**. Effect of different transition time points from apical growth to isotropic growth. Transition occurred when the bud height reached (A) $10L_{0}$. (B) $14L_{0}$. (C) $18L_{0}$. Once target heights were reached, the model was set to isotropically growth for an additional 40 simulation time steps. |

**SI: Tables**

| **Table A**. Mean bud radius and its standard deviation with respect to different expansion thresholds used. | | |
| --- | --- | --- |
| Expansion Threshold | Mean Bud Radius, $\mu m$ | Standard Deviation |
| $\gamma=0.025$ | 1.9154 | 0.0809 |
| $\gamma=0.075$ | 1.9145 | 0.0734 |
| $\gamma=0.1$ | 1.9131 | 0.0666 |
| $\gamma=0.125$ | 1.9149 | 0.0651 |

| **Table B:** Model parameters of the yeast mother cell | | | |
| --- | --- | --- | --- |
| Parameter | Description | Value(s) | Source |
| $k_{s}$ | Linear spring coefficient | 1.9 $\mu N/\mu m$ | Calibration using AFM data [(8)](https://www.zotero.org/google-docs/?lqUC20) |
| $k_{b}$ | Bending spring coefficient | 0.3 $\mu N\mu m$ | Calibration using AFM data [(8)](https://www.zotero.org/google-docs/?QhlC6N) |
| $k_{a}$ | Area expansion resistance coefficient | 1.0 $\mu N/\mu m$ | Calibration using AFM data [(8)](https://www.zotero.org/google-docs/?K0CVOA) |
| $k_{s}^{ring}$ | Linear spring coefficient, combined chitin and septin ring | 50.0 $\mu N\mu m$ | Model assumption based on qualitative observation. |
| $L_{0}$ | Initial edge length | 0.301 $\mu m$ | Relaxed initial system |
| $\theta_{0}$ | Initial dihedral angle | 0.08725 rad | Relaxed initial system |
| $L_{0}^{rep}$ | Volume exclusion length | 0.301 $\mu m$ | Qualitative observation |
| $D$ | Morse potential well depth | 0.01 | Qualitative observation |
| $a$ | Morse potential well width | 9.0 | Qualitative observation |
| $A_{0}$ | Initial triangular area | 0.03927 $\mu m^{2}$ | Relaxed initial system |
| $P$ | Turgor pressure | 0.2 MPa | [(11, 12)](https://www.zotero.org/google-docs/?JuPqLG) |
| $N_{relaxation}$ | Relaxation steps between the edge re-connectivity algorithm | 50 | Model assumption |

| **Table C**: Bud radii and the height from bud tip to the septin and chitin ring positions. Delayed repolarization (DR) indicates the height of the bud to reach before the biased growth occurs. Polarization height (PH) indicates the biased growth region. This region is determined by the height measured from the tip of the bud toward the mother cell. $R_{min}$ is the equilibrium length of the linear spring potential in the model. | | | |
| --- | --- | --- | --- |
| Budding condition | Mean total cell volume | Mean bud radii | Mean bud tip to the rings height |
| $DR=0, PH=2R_{min}$ | 53.7930 | 1.7926 | 5.5795 |
| $DR=0, PH=4R_{min}$ | 53.8033 | 1.6456 | 3.6556 |
| $DR=4R_{min}, PH=2R_{min}$ | 53.7902 | 1.7844 | 5.5021 |
| $DR=4R_{min}, PH=4R_{min}$ | 53.8092 | 1.6460 | 3.6883 |
| $DR=6R_{min}, PH=2R_{min}$ | 53.8315 | 1.7343 | 5.0569 |
| $DR=6R_{min}, PH=4R_{min}$ | 53.8134 | 1.6467 | 3.6430 |

| **Table D**: Bud radii and the height from bud tip to the septin and chitin ring positions. Delayed repolarization (DR) indicates the height of the bud to reach before anisotropic mechanical properties occur. K indicates the position of EC50 used in the Hill function such that $K=1$ implies EC50 is chosen at the septin and chitin ring positions and $K=0.5$ implies EC50 is chosen at the midpoint between bud tip and the ring positions. $n$ is the Hill coefficient. | | | |
| --- | --- | --- | --- |
| Budding condition | Mean total cell volume | Mean bud radii | Mean bud tip to the rings height |
| $K=1, n=8, DR=0$ | 53.8996 | 1.6568 | 3.7981 |
| $K=1, n=8, DR=4R_{min}$ | 53.8706 | 1.6528 | 3.7399 |
| $K=1, n=8, DR=6R_{min}$ | 53.8724 | 1.6526 | 3.6780 |
| $K=0.5, n=8, DR=0$ | Failed to bud | Failed to bud | Failed to bud |
| $K=0.5, n=8, DR=4R_{min}$ | 53.8956 | 1.6613 | 3.8151 |
| $K=0.5, n=8, DR=6R_{min}$ | 53.8356 | 1.6595 | 3.7988 |

| **Table E**: Bud radii and the height from bud tip to the septin and chitin ring positions. Delayed repolarization (DR) indicates the height of the bud to reach before anisotropic mechanical properties occur. K indicates the position of EC50 used in the Hill function such that $K=1$ implies EC50 is chosen at the septin and chitin ring positions and $K=0.5$ implies EC50 is chosen at the midpoint between bud tip and the ring positions. $n$ is the Hill coefficient. | | | |
| --- | --- | --- | --- |
| Budding condition | Mean total cell volume | Mean bud radii | Mean bud tip to the rings height |
| $K=1, n=16, DR=0$ | 53.8976 | 1.6501 | 3.5508 |
| $K=1, n=16, DR=4R_{min}$ | 53.8103 | 1.6454 | 3.5400 |
| $K=1, n=16, DR=6R_{min}$ | 53.7587 | 1.6458 | 3.5904 |
| $K=0.5, n=16, DR=0$ | Failed to bud | Failed to bud | Failed to bud |
| $K=0.5, n=16, DR=4R_{min}$ | 53.8547 | 1.6606 | 3.7029 |
| $K=0.5, n=16, DR=6R_{min}$ | 53.7458 | 1.6572 | 3.7615 |

**Local discontinuous Galerkin (LDG) method for solving reaction-diffusion equations (1) on a surface.** A LDG method developed in [(9)](https://www.zotero.org/google-docs/?zze5Kp) is utilized to solve model Eqs. (1), and it is briefly described below. We first fix notations. In the present work, we use a triangulated surface $\Gamma_{h}$ composed of planar triangles $K_{h}$ whose vertices stand on $\Gamma$ to approximate cell membrane $\Gamma$. Therefore, $\Gamma_{h}=\bigcup_{K_{h}\in T_{h}}K_{h}$, where $T_{h}$ denotes the set of the planar triangles that form an admissible triangulation. Denote by $E$ the set of edges (facets) of $T_{h}$. For each $e\in E$, denote by $h_{e}$ the length of the edge $e$. Let $N_{K_{h}}$ be an integer index of element $K_{h}$, and $K_{h}^{e,-}$ and $K_{h}^{e,+}$ be the two elements sharing the common edge $e$. Denote by $n_{h}^{-}$ and $n_{h}^{+}$ the unit outward conormal vectors defined on the edge $e$ for $K_{h}^{e,-}$ and $K_{h}^{e,+}$, respectively. The conormal $n_{h}^{-}$ to a point $x\in e$ is defined as follows according to [(10)](https://www.zotero.org/google-docs/?Qv0YBw) in SI References.

- 1. the unique unit vector that lies in the plane containing $K_{h}^{e,-}$;
  2. $n_{h}^{-}\left( x \right)\cdot\left( x-y \right)\geq0, \forall y\in K_{h}^{e,-}\cap B_{\epsilon}\left( x \right),$ where $B_{\epsilon}\left( x \right)$ is a ball centered in $x$. The radius $\epsilon(>0)$of $B_{\epsilon}\left( x \right)$ is sufficiently small so that $\epsilon<< |e|$, the length of edge $e$.

The conormal $n_{h}^{+}$ is defined similarly. With this definition, we have $n_{h}^{+}\neq n_{h}^{-}$ in general. Let $P_{k}(D)$ denote the space of polynomials of degree not greater than $k$ on any planar domain $D$. The discrete DG space $S_{h,k}$ of scalar function associated with $\Gamma_{h}$ is $S_{h,k}=\left\{ \chi\in L^{2}\left( \Gamma_{h} \right):\chi|_{K_{h}}\in P_{k}\left( K_{h} \right), \forall K_{h}\in T_{h} \right\},$ i.e., the space of piecewise polynomials which are globally in $L^{2}\left( \Gamma_{h} \right)$. The vector-valued DG space $\Sigma_{h,k}$ associated with $\Gamma_{h}$ is chosen to be $\Sigma_{h,k}=\left\{ \varphi\in\left[ L^{2}\left( \Gamma_{h} \right) \right]^{3}:\varphi|_{K_{h}}\in\left[ P_{k}\left( K_{h} \right) \right]^{3}, \forall K_{h}\in T_{h} \right\}.$ For $v_{h}\in S_{h,k}$ and $r_{h}\in\Sigma_{h,k}$, we use $v_{h}^{\pm}$ and $r_{h}^{\pm}$ to denote the trace of $v_{h}$ and $r_{h}$ on $e=K_{h}^{e,-}\cap K_{h}^{e,+}$ taken within the interior of $K_{h}^{e,-}$ and $K_{h}^{e,+}$, respectively. We refer readers to [(13)](https://www.zotero.org/google-docs/?4DAwiR) for the definitions of the surface gradient operator $\nabla_{\Gamma}$ and the Laplace-Beltrami operator $\Delta_{\Gamma}$ on $\Gamma$. By introducing the auxiliary variable $q=\sqrt{D}\nabla_{\Gamma}a$, the model problem Eqs. (1) can be rewritten as a first-order system of equations:

$a_{t}+\nabla_{\Gamma}\cdot\left( -\sqrt{D}q \right)=f(a) ,$

$q-\nabla_{\Gamma}g(a)=0 ,$

$\frac{db}{dt}=k_{4}\left( \bar{a}-k_{ss} \right)b ,$

$f\left( a \right)=\frac{k_{0}}{1+(\beta u)^{-q}}+\frac{k_{1}}{1+(\gamma pa)^{-h}}-k_{2}a - k_{3}ba , \bar{a}=\frac{\oint ads}{\oint1ds}, p=\frac{1}{1+(\beta u)^{-q}} .$

where $g\left( a \right)=\sqrt{D}a$. The semi-discrete LDG for solving the above equations is defined by: Find $a_{h}\in S_{h,k}$ and $q_{h}\in\Sigma_{h,k}$, such that for all test functions $v_{h}\in S_{h,k}$ and $r_{h}\in\Sigma_{h,k}$,

$\int_{K_{h}} \left( a_{h} \right)_{t}v_{h}dx-\int_{K_{h}} \left( -\sqrt{D}q_{h} \right)\cdot\nabla_{\Gamma_{h}}v_{h}dx-\int_{\partial K_{h}} \hat{\sqrt{D}q_{h}}\cdot n_{h}v_{h}dx=\int_{K_{h}} fv_{h}dx$

$\int_{K_{h}} q_{h}\cdot r_{h}dx=-\int_{K_{h}} g\left( a_{h} \right)\nabla_{\Gamma_{h}}\cdot r_{h}dx+\int_{\partial K_{h}} \hat{g}{n_{h}\cdot r}_{h}dx , (S1)$

$$\frac{db_{h}}{dt}=k_{4}\left( \underline{a}_{h}-k_{ss} \right)b_{h} ,$$

$$\underline{a}_{h}=\frac{\oint_{\Gamma_{h}} a_{h}ds}{\oint_{\Gamma_{h}} 1ds}, p=\frac{1}{1+(\beta u)^{-q}} .$$

Here $\hat{g}$ and $\hat{\sqrt{D}q_{h}}$ are numerical fluxes which will be described later on. To facilitate definitions of numerical fluxes, trace operators $\{\cdot\}$ and $⟦\cdot⟧$ are introduced by following ideas in [(10)](https://www.zotero.org/google-docs/?nVoJpd).

**Definition.** Denote by $K_{h}^{e,-}$ and $K_{h}^{e,+}$ the two elements sharing the common edge $e$. For $v\in L^{2}\left( \Gamma_{h} \right),$ $\{v\}$ and $⟦v⟧$ are defined as $\left\{ v \right\}=\frac{1}{2}\left( v^{-}+v^{+} \right), ⟦v⟧=v^{+}-v^{-}$ on $e$. For $\varphi\in\left[ L^{2}\left( \Gamma_{h} \right) \right]^{3}$, $\{\varphi,n_{h}\}$ and $⟦\varphi,n_{h}⟧$ are defined as $\left\{ \varphi,n_{h} \right\}=\frac{1}{2}\left( \varphi^{+}\cdot n_{h}^{+}-\varphi^{-}\cdot n_{h}^{-} \right), ⟦\varphi,n_{h}⟧=\varphi^{+}\cdot n_{h}^{+}+\varphi^{-}\cdot n_{h}^{-}$ on $e$.

Denote by $S_{K_{h}^{+}}^{K_{h}^{-}}\in\{0,1\}$ a switch function [(14)](https://www.zotero.org/google-docs/?SFpeqT). $S_{K_{h}^{+}}^{K_{h}^{-}}$ is associated with $K_{h}^{+}$ on the edge that $K_{h}^{+}$ and $K_{h}^{-}$ share, and is defined by:

$$S_{K_{h}^{+}}^{K_{h}^{-}}=\{1 , if N_{K_{h}^{+}}>N_{K_{h}^{-}} ; 0 , otherwise.$$

The numerical fluxes on the edge $e$ for $K_{h}^{e,-}$ and $K_{h}^{e,+}$ are defined respectively, as follows. The diffusive fluxes $\left( {\hat{\sqrt{D}q}}^{+}, \hat{g}^{+} \right)^{T}$ and $\left( {\hat{\sqrt{D}q}}^{-}, \hat{g}^{-} \right)^{T}$:

$${\hat{\sqrt{D}q}}^{+}=\left( \frac{⟦g\left( a_{h} \right)⟧}{⟦a_{h}⟧}\left\{ q_{h},n_{h} \right\}-C_{11}⟦a_{h}⟧+C_{12}\cdot n_{h}^{+}⟦q_{h},n_{h}⟧ \right)n_{h}^{+},$$

$${\hat{\sqrt{D}q}}^{-}=-\left( \frac{⟦g\left( a_{h} \right)⟧}{⟦a_{h}⟧}\left\{ q_{h},n_{h} \right\}-C_{11}⟦a_{h}⟧+C_{12}\cdot n_{h}^{+}⟦q_{h},n_{h}⟧ \right)n_{h}^{-};$$

$$\hat{g}^{+}=\left\{ g\left( a_{h} \right) \right\}-C_{12}\cdot n_{h}^{+}⟦a_{h}⟧,$$

$$\hat{g}^{-}=\left\{ g\left( a_{h} \right) \right\}-C_{12}\cdot n_{h}^{+}⟦a_{h}⟧.$$

Here the penalization coefficients $C_{11}$ and $C_{12}$ are chosen to be

$$C_{11}=\frac{1}{h_{e}}, C_{12}=\frac{1}{2}\left( S_{K_{h}^{e,+}}^{K_{h}^{e,-}}n_{h}^{+}+S_{K_{h}^{e,-}}^{K_{h}^{e,+}}n_{h}^{-} \right).$$

With the above choices of the numerical fluxes, it yields that $⟦\hat{g}⟧=0$ and $⟦\hat{\sqrt{D}q,}n⟧=0$. Thus, these numerical fluxes are consistent and conservative. Moreover, they allow for a local resolution of $q_{h}$ in terms of $a_{h}$. The second-order accurate TVD Runge-Kutta (RK) time discretization is used to solve the semi-discrete scheme $(S1)$, which can be formulated as an ordinary differential equation:

$\Phi_{t}=L\left( \Phi,t \right). (S2)$

The second-order accurate TVD RK method for solving Eq. $(S2)$ is given by

$\Phi^{(1)}=\Phi^{n}+\Delta t_{n}L(\Phi^{n},t_{n}) , \Phi^{n+1}=\frac{1}{2}\Phi^{n}+\frac{1}{2} \Phi^{(1)}+\frac{1}{2}\Delta t_{n}L\left( \Phi^{\left( 1 \right)},t_{n+1} \right).$

Here $\Delta t_{n}$ is the time step size. In this paper, we only considered planar triangulations of the surface which are at most second-order accurate. Therefore, we choose the second-order accurate TVD RK time-stepping method. Also, the polynomial degree $k$ of the DG spaces used in this work is 1. A higher-order accurate surface approximation is needed to improve the overall accuracy of the scheme. We refer readers to [(9)](https://www.zotero.org/google-docs/?isB6yU) for accuracy tests of this numerical scheme.

**SI References.**

[1. M. Jin, *et al.*, Divergent Aging of Isogenic Yeast Cells Revealed through Single-Cell Phenotypic Dynamics. *Cell Syst* **8**, 242-253.e3 (2019).](https://www.zotero.org/google-docs/?fKwhmN)

[2. Y. Li, *et al.*, A programmable fate decision landscape underlies single-cell aging in yeast. *Science* **369**, 325–329 (2020).](https://www.zotero.org/google-docs/?fKwhmN)

[3. Z. Zhou, *et al.*, Engineering longevity—design of a synthetic gene oscillator to slow cellular aging. *Science* **380**, 376–381 (2023).](https://www.zotero.org/google-docs/?fKwhmN)

[4. Y. Li, *et al.*, Multigenerational silencing dynamics control cell aging. *Proc Natl Acad Sci U A* **114**, 11253–11258 (2017).](https://www.zotero.org/google-docs/?fKwhmN)

[5. C.-S. Chou, Q. Nie, T.-M. Yi, Modeling Robustness Tradeoffs in Yeast Cell Polarization Induced by Spatial Gradients. *PLoS ONE* **3**, e3103 (2008).](https://www.zotero.org/google-docs/?fKwhmN)

[6. K. Tsai, *et al.*, Role of combined cell membrane and wall mechanical properties regulated by polarity signals in cell budding. *Phys Biol* **17**, 065011 (2020).](https://www.zotero.org/google-docs/?fKwhmN)

[7. K. D. Moran, D. J. Lew, How Diffusion Impacts Cortical Protein Distribution in Yeasts. *Cells* **9**, 1113 (2020).](https://www.zotero.org/google-docs/?fKwhmN)

[8. E. Dague, *et al.*, An atomic force microscopy analysis of yeast mutants defective in cell wall architecture. *Yeast* **27**, 673–684 (2010).](https://www.zotero.org/google-docs/?fKwhmN)

[9. S. Xu, Z. Xu, Local Discontinuous Galerkin Methods for Solving Convection-Diffusion and Cahn-Hilliard Equations on Surfaces (2024) https:/doi.org/10.48550/ARXIV.2401.02069 (February 16, 2024).](https://www.zotero.org/google-docs/?fKwhmN)

[10. P. F. Antonietti, *et al.*, High Order Discontinuous Galerkin Methods for Elliptic Problems on Surfaces. *SIAM J. Numer. Anal.* **53**, 1145–1171 (2015).](https://www.zotero.org/google-docs/?fKwhmN)

[11. B. Goldenbogen, *et al.*, Dynamics of cell wall elasticity pattern shapes the cell during yeast mating morphogenesis. *Open Biol.* **6**, 160136 (2016).](https://www.zotero.org/google-docs/?fKwhmN)

[12. J. Schaber, *et al.*, Biophysical properties of Saccharomyces cerevisiae and their relationship with HOG pathway activation. *Eur. Biophys. J.* **39**, 1547–1556 (2010).](https://www.zotero.org/google-docs/?fKwhmN)

[13. G. Dziuk, C. M. Elliott, Finite element methods for surface PDEs. *Acta Numer.* **22**, 289–396 (2013).](https://www.zotero.org/google-docs/?fKwhmN)

[14. J. Peraire, P.-O. Persson, The Compact Discontinuous Galerkin (CDG) Method for Elliptic Problems. *SIAM J. Sci. Comput.* **30**, 1806–1824 (2008).](https://www.zotero.org/google-docs/?fKwhmN)
